# A role for curcumin in preventing liver fibrosis in animals: a systematic review and meta-analysis

**DOI:** 10.3389/fphar.2024.1396834

**Published:** 2024-05-24

**Authors:** Bo-Hao Huang, Zi-Wei Guo, Bo-Han Lv, Xin Zhao, Yan-Bo Li, Wen-Liang Lv

**Affiliations:** ^1^Department of Infection, Guang’an Men Hospital, China Academy of Chinese Medical Sciences, Beijing, China; ^2^Graduate school, Beijing University of Chinese Medicine, Beijing, China

**Keywords:** Chinese medicine, hepatic fibrosis, curcumin against hepatic fibrosis, preclinical meta-study, experiments on animals

## Abstract

**Objective:**

This meta-analysis aimed to determine the efficacy of curcumin in preventing liver fibrosis in animal models.

**Methods:**

A systematic search was conducted on studies published from establishment to November 2023 in PubMed, Web of Science, Embase, Cochrane Library, and other databases. The methodological quality was assessed using Sycle’s RoB tool. An analysis of sensitivity and subgroups were performed when high heterogeneity was observed. A funnel plot was used to assess publication bias.

**Results:**

This meta-analysis included 24 studies involving 440 animals with methodological quality scores ranging from 4 to 6. The results demonstrated that curcumin treatment significantly improved Aspartate aminotransferase (AST) [standard mean difference (SMD) = -3.90, 95% confidence interval (CI) (−4.96, −2.83), *p* < 0.01, I^2^ = 85.9%], Alanine aminotransferase (ALT)[SMD = − 4.40, 95% CI (−5.40, −3.40), *p* < 0.01, I^2^ = 81.2%]. Sensitivity analysis of AST and ALT confirmed the stability and reliability of the results obtained. However, the funnel plot exhibited asymmetry. Subgroup analysis based on species and animal models revealed statistically significant differences among subgroups. Furthermore, curcumin therapy improved fibrosis degree, oxidative stress level, inflammation level, and liver synthesis function in animal models of liver fibrosis.

**Conclusion:**

Curcumin intervention not only mitigates liver fibrosis but also enhances liver function, while concurrently modulating inflammatory responses and antioxidant capacity in animal models. This result provided a strong basis for further large-scale animal studies as well as clinical trials in humans in the future.

**Systematic Review Registration:** https://www.crd.york.ac.uk/prospero/, identifier CRD42024502671.

## 1 Introduction

Hepatic fibrosis arises from an imbalanced reparative response to chronic injury, and its progression may result in cirrhosis, liver failure, hepatocellular carcinoma, and other severe conditions (Hernandez-Gea and Friedman, 2011; Ginès et al., 2021). This condition is marked by an abnormal accumulation and irregular distribution of extracellular matrix (ECM) components, including collagen and glycoproteins, disrupting the normal functioning of liver cells. Such disruption triggers myofibroblast activation, leading to structural disorders and the loss of liver function (Asrani et al., 2019). Excessive production of ECM destroys liver structure, impairing organ function, disrupting blood flow, and potentially causing cirrhosis (Y. Wang et al., 2023).

**FIGURE 1:**
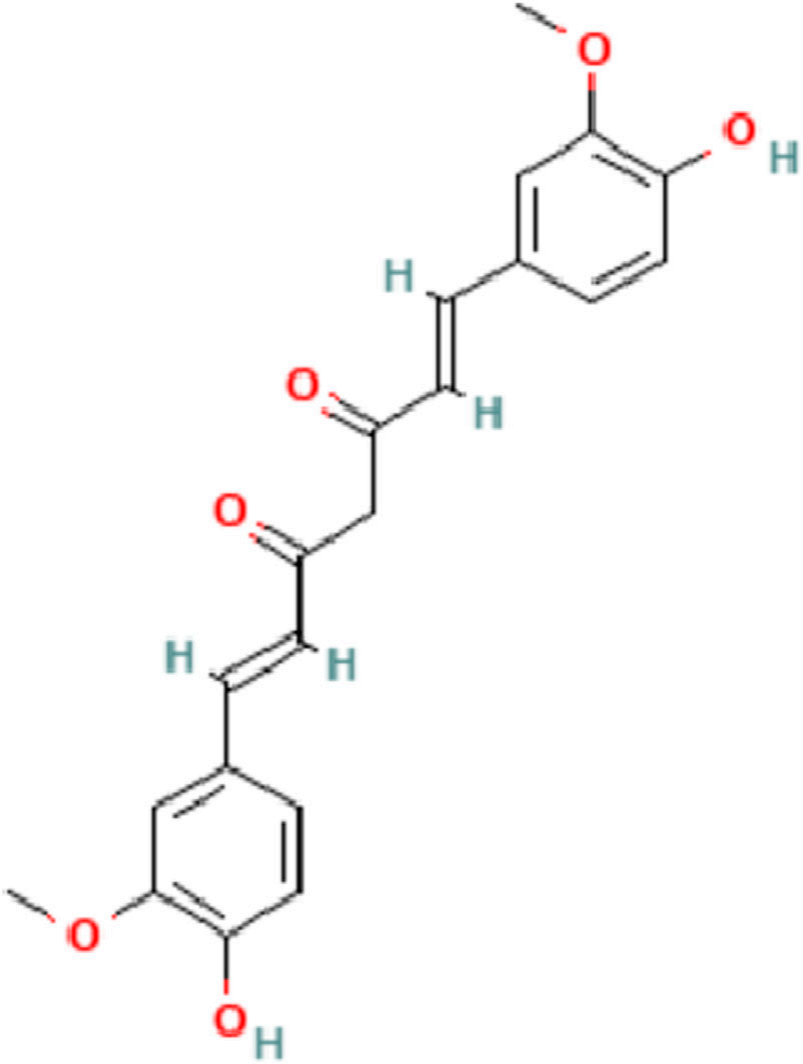
The chemical structure of Curcumin.

In 2017, the global prevalence of chronic liver disease reached 1.5 billion cases, with non-alcoholic fatty liver disease (NAFLD), hepatitis B virus (HBV) infection, hepatitis C virus (HCV) infection, and alcoholic liver disease (ALD) constituting approximately 60%, 29%, 9%, and 2% of cases, respectively (James et al., 2018; Moon, Singal, and Tapper, 2020). Liver fibrosis represents the inevitable stage in the development of all chronic liver diseases. Anti-fibrotic interventions play a crucial role in delaying the progression of liver disease and enhancing the quality of life for patients. However, progress in the research on anti-fibrosis drugs is sluggish at present. Conventional antiviral, anti-inflammatory therapies and other treatment methods employed in the treatment of liver fibrosis have proven ineffective in ECM deposition or facilitating its degradation (Ma et al., 2020). As a result, they are unable to adequately manage or eliminate liver fibrosis. Over an extended period, both domestically and internationally, significant attention has been directed toward the pharmacological properties and clinical development of curcumin (Sun and Kisseleva, 2015). Curcumin, derived from turmeric, has long captivated the pharmaceutical field globally due to its pharmacological effects and clinical development (Hasanzadeh et al., 2020). Curcumin is a lipophilic polyphenol that is antioxidant, anti-inflammatory, and anti-fibrotic. It has been extensively used as a food seasoning because of its high level of safety (Bavarsad et al., 2019) (Figure 1). Numerous human clinical trials have extensively employed curcumin for interventions in various diseases, such as multiple myeloma, pancreatic cancer, NAFLD, colon cancer, and Alzheimer’s disease, both *in vitro* and in animal models (Hatcher et al., 2008).

A growing body of evidence suggests that curcumin also holds therapeutic potential for liver fibrosis. It regulates cytokine production by regulating several signaling pathways, including those involved in inflammation. Including the transforming growth factor-beta (TGF-β)/Smad Signaling Pathway (N. Chen et al., 2014), PPAR-γ Signaling Pathway (Jin et al., 2016), NF-κB pathway (Cui et al., 2014), etc. This regulation inhibits hepatic stellate cell (HSC) activation while reducing liver inflammation and tissue oxidative stress levels (Y. Tang, 2015). Therefore, curcumin appears to be a highly effective treatment for liver fibrosis. However, no systematic review on this topic exists. Consequently, we conducted a meta-analysis based on preclinical data to evaluate the efficacy of curcumin, enhancing the credibility of the evidence, fortifying this conclusion, and providing clinical guidance for patients with hepatic fibrosis.

## 2 Methods

The systematic review and meta-analysis were conducted following the guidelines outlined in the Preferred Reporting Items for Systematic Reviews and Meta-Analysis (PRISMA).

### 2.1 Search strategy

The systematic search was conducted in four databases—PubMed, Web of Science, Embase, and Cochrane Library—from the establishment to October 2000 to November 2023, confined to English, Primary keywords included ‘liver cirrhosis’, ‘Hepatic Cirrhosis’, ‘Cirrhosis, Hepatic’, as well as ‘Curcumin’, ‘Turmeric Yellow’, ‘Yellow, Turmeric’, and ‘Curcumin Phytosome’. The comprehensive electronic search strategy for all databases is provided in Supplementary S1.

### 2.2 Inclusion and exclusion criteria

The predetermined inclusion criteria were as follows: 1) papers: describing the number of animals used; 2) language: published in English-language journals; 3) participants: liver fibrosis animal models; 4) intervention: Curcumin as the sole intervention; 5) comparison: a placebo solution or no treatment; 6) primary outcomes: AST and ALT.

Excluded documents criteria were as follows:1) clinical studies, reviews; 2) vitro studies; 3) conference reports and comments; 4) unpublished or duplicate literature; 5) lacking a control group.

### 2.3 Data extraction

Following predefined inclusion and exclusion criteria, we reviewed both abstracts and full texts of papers to establish the final selection of research literature. Any discrepancies regarding the eligibility of a specific study were resolved through discussions with a third-party reviewer.

Standardized pre-test tables in Excel were employed for data extraction from the included studies to facilitate evidence synthesis. The extracted information encompassed the following aspects:

(a) The first author’s name and the year of publication of the document;
(b) Species, sex, and weight of the animal model, along with the sample size for each group;
(c) Methods employed for modeling liver fibrosis;
(d) Dose (with a focus on the maximum dose when different doses were utilized), time, and duration of administration in the treatment group.

In cases where study data was presented graphically, efforts were made to obtain the original numerical values from the authors. If raw data is not available, use the icon Digital Ruler software to measure the icon data (GetData Graph Digitizer 2.26, https://www.softpedia.com/get/Multimedia/Graphic/Graphic-Others/GetData-Graph-Digitizer.shtml). If the data provided in the text shows SE, convert it to SD, using the formula: SD = SEM* $\sqrt{n}$ .

### 2.4 Risk of bias assessment

To assess the data quality in the articles included in this review, two researchers (Yan-bo Li and Zi-wen Zhuo) employed SYRGLE’s bias risk tool (Hooijmans et al., 2014). The assessment comprehensively addresses ten bias areas, including those related to selection, detection, performance, attrition, reporting bias, and other biases. In cases of disagreement, a third researcher was consulted. The tool provides three possible responses: "+" signifies a low risk of bias, "-" indicates a high risk of bias, and "?" denotes that the representation concept cannot be definitively attributed.

### 2.5 Statistical analysis

Summary statistics were assessed using the SMD and 95% CI. The heterogeneity of the studies was evaluated using the I^2^ statistics. Based on the level of heterogeneity, different effect models were employed. The random effect model was utilized when I^2^ >50%, while the fixed effect model was employed otherwise. Stata 16.0 was used for all statistical analyses.

The source of heterogeneity was identified through subgroup analysis and sensitivity analysis. When the number of included studies exceeded 10, publication bias was assessed using a funnel plot. A significance level of *p* < 0.05 indicated a statistically significant difference.

## 3 Results

### 3.1 Study selection

**FIGURE 2:**
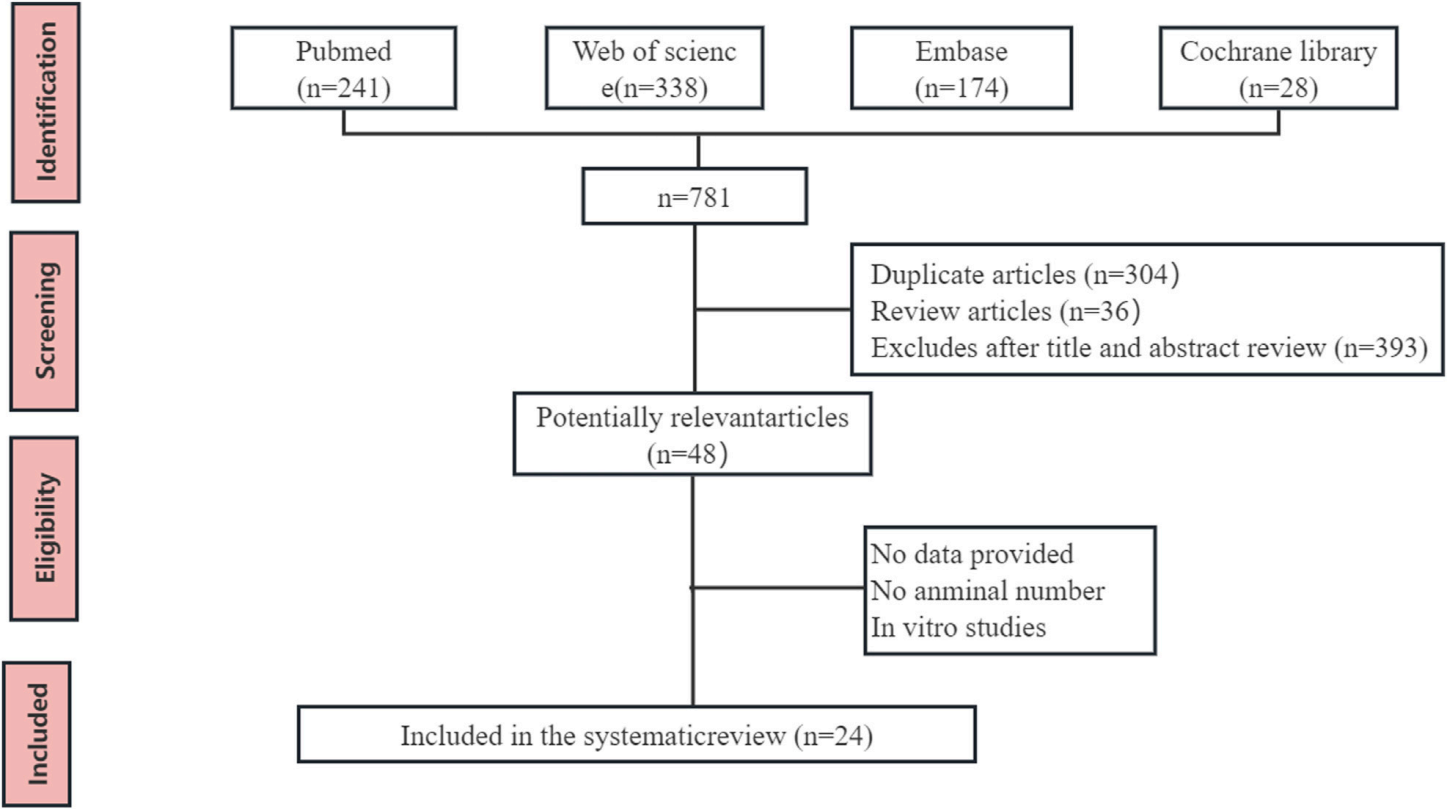
Flow diagram of database searches and study selection.

A total of 735 articles were initially identified. After removing duplicates and reviewing articles, the remaining studies underwent screening based on titles and abstracts, leading to the exclusion of 687 articles. Subsequently, two independent researchers (Bo-hao Huang and Bo-han Lv) meticulously reviewed the full text of 48 articles, evaluating them against the predetermined eligibility criteria. In total, 24 studies involving 440 animals were included in this meta-analysis. The flow chart of database search and research selection is shown in Figure 2.

### 3.2 Characteristics of the included studies

Table 1 summarizes key characteristics of the reviewed literature, encompassing: 1) first author; 2) year of publication; 3) species and sex of the animals; 4) weight; 5) number of subjects in each group; 6) modeling method; 7) curcumin dose, route, and duration; 8) histopathology.

**TABLE 1:** Characteristics of the included studies.

First author et al., year	Species, sex	Weight	n = experimental/model group	Model (method)	Treatment group (method)	Liver histopathology	Outcome index
Gowifel et al., 2020	Wistar rats; male	150–170g	8/8	subcutaneous injection; TAA (100 mg/kg); three times a week, for 8 weeks	curcumin (200 mg/kg, p.o.); daily, for 8 weeks	Masson’s trichrome; H&E Staining	①②④⑧⑫⑬⑭⑱
Macías-Pérez et al., 2019	*Mesocricetus auratus*; male	100–150g	5/5	intraperitoneal injection; CCI4(50 mg/kg, dissolved in petrolatum), two times a week, for 20 weeks	curcumin (30 mg/kg, p.o.); daily, for 4 weeks	H&E Staining; Sirius red Staining	①②④⑤⑥⑨⑩⑯
El et al. (2016)	Wister rats; male	180–220g	8/8	surgical ligation of the common bile duct	curcumin (20 mg/kg, p.o.); daily, for 2 weeks	Masson’s trichrome; H&E Staining	①②⑨⑩⑫⑯⑱
Wu et al. (2010)	Sprague-Dawley rats; male	200–250g	10/10	intraperitoneal injection; CCI4 (0.75 mL/kg,40% in olive oil); once a week, for 7 weeks	curcumin (0.005%,p.o.); daily, for 8 weeks	Sirius Red Staining	⑨
Eshaghian et al., 2018	Wistar rats; male	200–250g	8/8	surgical ligation of the common bile duct	curcumin (100 mg/kg, p.o.); daily, for 4 weeks	H&E Staining	①②③⑧⑩⑬
Hernández-Aquino et al. (2020)	Wistar rats; male	100–120g	8/8	intraperitoneal injection; CCI4 (400 mg/kg); three times a week, for 12 weeks	curcumin (100 mg/kg, p.o.); tiwce a day, for 4 weeks	H&E Staining; Masson’s trichrome	②⑦⑰⑱
Khodarahmi et al. (2020)	Wistar rats; male	200–250g	8/8	surgical ligation of the common bile duct	curcumin (100 mg/kg, p.o.); daily, for 4 weeks	H&E Staining	①②⑧⑬
Zhang et al. (2013)	Sprague-Dawley rats; male	180–220g	6/6	intraperitoneal injection; CCI4 (0.1 mL/100g, olive oil 1:1 (w/v)); once every other day, for 8 weeks	curcumin (dose not clear, p.o.); daily, for 4 weeks	H&E Staining; Masson’s trichrome	⑪⑭
Wu et al. (2008)	Sprague-Dawley rats; male	200–250g	10/10	intraperitoneal injection; 40% CCI4 (0.75 mL/kg); once a week, for 8 weeks	curcumin (0.005%,p.o.); daily, for 8 weeks	H&E Staining; Masson’s trichrome	①②
Reyes-Gordillo et al., 2008	Wistar rats; male	200–250g	4–5/4–5	bile duct ligation	curcumin (100 mg/kg, p.o.); daily, for 8 weeks	H&E Staining; Masson’s trichrome	②⑦⑰⑱
Zhao et al. (2014)	Sprague-Dawley rats; male	180–200g	10/10	intraperitoneal injection; CCI4(500 μL/100 g, mixed 1:1 with olive oil) for the first time and following at a dose of 300 μL/100 g (mixed 3:7 with olive oil); twice a week, for 6 weeks	curcumin (1200 mg/kg, p.o.); daily, for 6 weeks	H&E Staining	①②③④⑤⑥⑧
Qin et al. (2018)	Sprague-Dawley rats; male	200–220g	10/10	subcutaneous injection; 1.5 mL of CCI4/olive oil (2:3, v/v)/kg; every 3 days, for 8 weeks	curcumin (200 mg/kg, p.o.); daily for 8 weeks	H&E staining; Masson’s trichrome staining	①②④⑮⑯
Lu et al. (2017)	ICR mice; male	20–25g	12/12	subcutaneous injection; 1 mL of CCI4/olive oil (1:1, v/v)/kg; every other day, for 4 weeks	curcumin (400 mg/kg, p.o.), daily for 4 weeks	H&E Staining; Masson’s trichrome staining	①②⑮
Tu et al. (2012)	Sprague–Dawley rats; male	180–220g	10/10	intraperitoneal injection; CCI4(0.2 mL/kg); twice weekly, for 6 weeks	curcumin (200 mg/kg, p.o.); twice weekly for 6 weeks	H&E Staining; Sirius red	①②⑭
Abd-Allah et al. (2016)	Wistar rats; male	79–140g	9/9	intraperitoneal injection; CCI4(0.5 mL/kg mixed (v/v) in olive oil); twice weekly for 8 weeks	curcumin (200 mg/kg, p.o.); twice weekly for 8 weeks	H&E Staining; Sirius red	④⑤⑥⑫⑱
Morsy et al. (2012)	Wistar rats; male	180–200g	10/10	intraperitoneal injection; CCI4(olive oil (1:1, v/v,1 mL/kg); twice weekly for 8 weeks	curcumin (200 mg/kg, p.o.); twice weekly for 8 weeks	H&E Staining; Masson’s trichrome staining	①②⑪⑱
Lee et al. (2016)	Sprague-Dawley rats; male	250–270g	10/10	intraperitoneal injection; CCI4 (0.1 mL/100 g body weight, olive oil [1:1 (v/v)] every other day for 4 weeks	curcumin (200 mg/kg, p.o.); daily, for 4 weeks	Sirius red	①②⑱
Fu et al. (2008)	Sprague-Dawley rats; male	250–300g	6/6	intraperitoneal injection; CCI4(0.1mL/100g, mixed in olive oil1:1 (v/v)), every other day for 8weeks	curcumin (400 mg/kg, p.o.); daily, for 8 weeks	H&E Staining; Sirius red	①②③⑧⑱⑲
Zhao et al. (2018)	C57/B6 mice; male	20–22g	10/10	intraperitoneal injection; CCI4 (CCI4: olive oil = 1:4, 3 μL/g CCI4 oil); twice weekly for 4 weeks	curcumin (200 mg/kg, p.o.); twice weekly for 4 weeks	H&E Staining; Masson’s trichrome staining	①⑪
Kabirifar et al. (2018)	Wistar rats; male	200g	8/8	surgical ligation of the common bile duct	curcumin (200 mg/kg, p.o.); daily, for 4 weeks	H&E Staining	③⑧⑩⑲
Barta et al. (2015)	Wistar rat; male	unclear	8/8	surgical ligation of the common bile duct	curcumin (200 mg/kg, p.o.); daily, for 3 weeks	H&E Staining	⑤⑥
George et al., 2006	Wistar rat; male	150g	6/6	Intraperitoneal injection; N-nitrosodimethyl amine (1 mg/100g); 7consecutive days	curcumin (200 mg/kg, p.o.); 7 consecutive days	—	①②
Kyung et al. (2018)	Sprague-Dawley rats; male	260–280 g	6/6	intraperitoneal injection; N-nitrosodimethyl amine (10 μg/kg, dissolved in PBS); twice a week for 4 weeks	curcumin (100 mg/kg, p.o.); twice weekly for 4 weeks	H&E Staining	①②
Abo-Zaid et al. (2020)	*Rattus rattus* L. rats; male	unclear	12/12	Intraperitoneal injection; CCI4 (3 mL/kg); twice weekly for 6 weeks	curcumin (250 mg/kg, p.o.); twice weekly for 6 weeks	H&E Staining	①②
Yao et al., 2012	Sprague–Dawley rats; male	180–220g	10/15	intraperitoneal injection; CCI4 (CCI4/olive oil (1:1, v/v)/kg); twice weekly for 6 weeks	curcumin (200 mg/kg, p.o.), twice weekly for 6 weeks	H&E Staining; Sirius red	⑨

① AST; ② ALT; ③ ALP; ④ ALB; ⑤ TP; ⑥ Tbil; ⑦ γ-GTP; ⑧ mRNA, expression levels of α-SMA; ⑨ mRNA, expression levels of TGF-β; ⑩ mRNA, expression levels of NF-κb; ⑪ TG; ⑫ MDA; ⑬ collagen I; ⑭ SOD; ⑮ LN; ⑯ area of fibrosis; ⑰ collagen; ⑱ GSH; ⑲ HYP. p.o.: oral administration; TAA: thioacetamide; H&E: hematoxylin and eosin.

In terms of species, the studies involved Sprague-Dawley rats (9 studies), Wistar rats (11 studies), C57 BL/6 mice (1 study), *Rattus rattus* mice (1 study), ICR mice (1 study), and *Mesocricetus auratus* mice (1 study). All studies exclusively used male subjects. The methods employed for establishing animal models of liver fibrosis included CCI4 injection (15 studies), BDL surgery (5 studies), NDMA injection (3 studies), and TAA injection (1 study).

To assess the therapeutic impact of curcumin on liver fibrosis, we analyzed data from 17 studies that reported AST levels, 18 studies that reported ALT levels, 4 studies that reported alkaline phosphatase (ALP) levels, 5 studies that reported albumin (ALB) levels, 6 studies that reported total bilirubin (TBil) levels, 3 studies that reported collagen I levels, 5 studies that reported mRNA of alpha-smooth muscle actin (α-SMA) levels, 3 studies that reported malondialdehyde (MDA) levels, 3 studies that reported mRNA of TGF-β mRNA, 4 studies that reported mRNA of nuclear factor κB (NF-κb) levels, 3 studies that reported triglyceride (TG) levels, 3 studies that reported superoxide dismutase (SOD) levels, 8 studies that reported Glutathione (GSH) levels, 2 studies that reported Hydroxyproline (HYP) levels. 3 studies that reported laminin (LN) levels, and 3 studies that reported the degree of liver fibrosis.

### 3.3 Quality of the included studies

The quality of the literature included in the study underwent evaluation, with two researchers (Yanbo Li and Ziwen Zhuo) conducting a case-by-case assessment. The results revealed that the scores of the included literature varied between 4 and 6. Specifically, 15 studies scored 6 (Gowifel et al., 2020; Wu et al., 2010; Eshaghian et al., 2018; Khodarahmi et al., 2020; Y. Zhao et al., 2014; Qin et al., 2018; Lu et al., 2017; Chen et al., 2014; Wu et al., 2008; Morsy et al., 2012; Zhao et al., 2018; Kabirifar et al., 2018; Barta et al., 2015; Kyung et al., 2018), 8 studies scored 5 (Macías-Pérez et al., 2019; El et al., 2016; Hernández-Aquino et al., 2020; Zhang et al., 2013; Reyes-Gordillo et al., 2008; Fu et al., 2008; George et al., 2006; Abo-Zaid, Shaheen, and Ismail, 2020), and 1 study scored 4 (H.-Y. Lee et al., 2016).

Nineteen studies reported utilizing random assignment of animals, although specific methods of randomization were not mentioned. The remaining five studies did not mention animal randomization. Of the total 19 studies, it was indicated that the animals were maintained under identical conditions, while the remaining five studies did not provide information on the conditions of the animals. All the included literature reported complete data.

In terms of methodology, each study detailed the approach taken to balance inter-group baseline characteristics, and there was no evidence of selective reporting. None of the studies reported hidden assignment, blind intervention, randomization of outcome evaluation, or outcome blindness. Furthermore, no other sources of bias were identified across all studies. Table 2 provides a comprehensive assessment of the methodological quality of the included studies.

**TABLE 2:** The methodological quality of included studies.

(First author et al., year)	A	B	C	D	E	F	G	H	I	J	Score
Gowifel et al., 2020	+	+	-	+	-	-	?	+	+	+	6
Macías-Pérez et al., 2019	?	+	-	+	-	-	?	+	+	+	5
El et al. (2016)	?	+	-	+	-	-	?	+	+	+	5
Wu et al. (2010)	+	+	-	+	-	-	?	+	+	+	6
Eshaghian et al., 2018	+	+	-	+	-	-	?	+	+	+	6
Hernández-Aquino et al., 2020	?	+	-	+	-	-	?	+	+	+	5
Khodarahmi et al., 2020	+	+	-	+	-	-	?	+	+	+	6
Zhang et al. (2013)	?	+	-	+	-	-	?	+	+	+	5
Wu et al. (2008)	+	+	-	+	-	-	?	+	+	+	6
Reyes-Gordillo et al., 2008	+	+	-	?	-	-	?	+	+	+	5
Zhao et al. (2014)	+	+	-	+	-	-	?	+	+	+	6
Qin et al. (2018)	+	+	-	+	-	-	?	+	+	+	6
Lu et al. (2017)	+	+	-	+	-	-	?	+	+	+	6
Tu et al. (2012)	+	+	-	+	-	-	?	+	+	+	6
Abd-Allah et al. (2016)	+	+	-	+	-	-	?	+	+	+	6
Morsy et al., 2012	+	+	-	+	-	-	?	+	+	+	6
Lee et al. (2016)	?	+	-	?	-	-	?	+	+	+	4
Fu et al. (2008)	+	+	-	?	-	-	?	+	+	+	5
Zhao et al. (2018)	+	+	-	+	-	-	?	+	+	+	6
Kabirifar et al. (2018)	+	+	-	+	-	-	?	+	+	+	6
Barta et al. (2015)	+	+	-	+	-	-	?	+	+	+	6
George et al., 2006	+	+	-	?	-	-	?	+	+	+	5
Kyung et al. (2018)	+	+	-	+	-	-	?	+	+	+	6
Abo-Zaid et al. (2020)	+	+	-	?	-	-	?	+	+	+	5

A. sequence generation; B. baseline characteristics; C. allocation concealment D. random housing; E. blinding (caregivers/investigators) F. random for outcome assessment; G. blinding (outcome assessor) H. incomplete outcome data; I. selective outcome reporting; J. other biases.

### 3.4 Outcome measures

#### 3.4.1 Liver function

##### 3.4.1.1 AST

**FIGURE 3:**
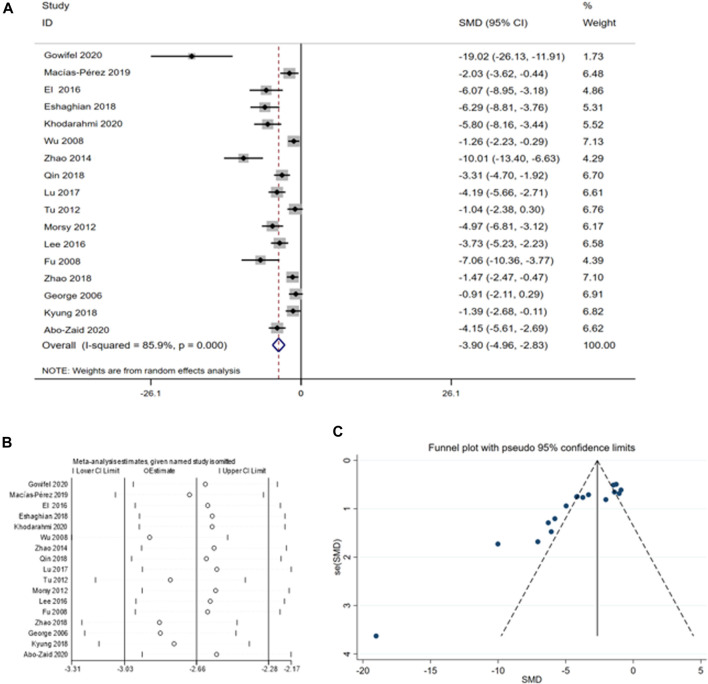
**(A)** Forest plot: antifibrosis effect of curcumin on AST; **(B)** sensitive analysis of AST; **(C)** funnel plot of AST.

This meta-analysis comprised 17 studies involving 284 animals to assess the impact of curcumin on AST levels (Gowifel et al., 2020; Macías-Pérez et al., 2019; Wu et al., 2010; Eshaghian et al., 2018; Khodarahmi et al., 2020; Wu et al., 2008; Zhao et al., 2014; Qin et al., 2018; Lu et al., 2017; Tu et al., 2012; Morsy et al., 2012; Lee et al., 2016; Fu et al., 2008; Zhao et al., 2018; George et al., 2006; Kyung et al., 2018; Abo-Zaid, Shaheen, and Ismail, 2020). The summary analysis indicated a significant reduction in AST levels with curcumin compared to the CCI4 model group [SMD = −3.90, 95% CI (−4.96, −2.83), *p* < 0.01, I^2^ = 85.9%, Figure 3A].

A thorough analysis was performed because of the high heterogeneity of the sample, including sensitivity analysis, funnel plot examination, and subgroup analysis. According to the sensitivity analysis, omitting individual studies did not affect the aggregated estimates of AST significantly (Figure 3B). However, the funnel plot for AST displayed asymmetry (Figure 3C), we examined the sources of heterogeneity using subgroup analysis based on species (Supplementary Figure S1) and animal models (Supplementary Figure S2) (Table 3).

**TABLE 3:** Subgroup analysis of AST.

Subgroup	Number of paper	SMD	95% CI	*p*-value	Heterogeneity
Species					
Wistar rats	6	−6.06	−9.03, −3.08	*p* < 0.01	90.0
Sprague-Dawley rats	7	−3.40	−4.98, −1.82	*p* < 0.01	86.1
Method for molding					
CCI 4	11	−3.50	−2.47, −0.47	*p* < 0.01	83.7
BDL	4	−6.04	−7.52, 4.56	*p* = 0.962	0
NDMA	2	−1.13	−2.01, −0.26	*p* = 0.59	0

##### 3.4.1.2 ALT

**FIGURE 4:**
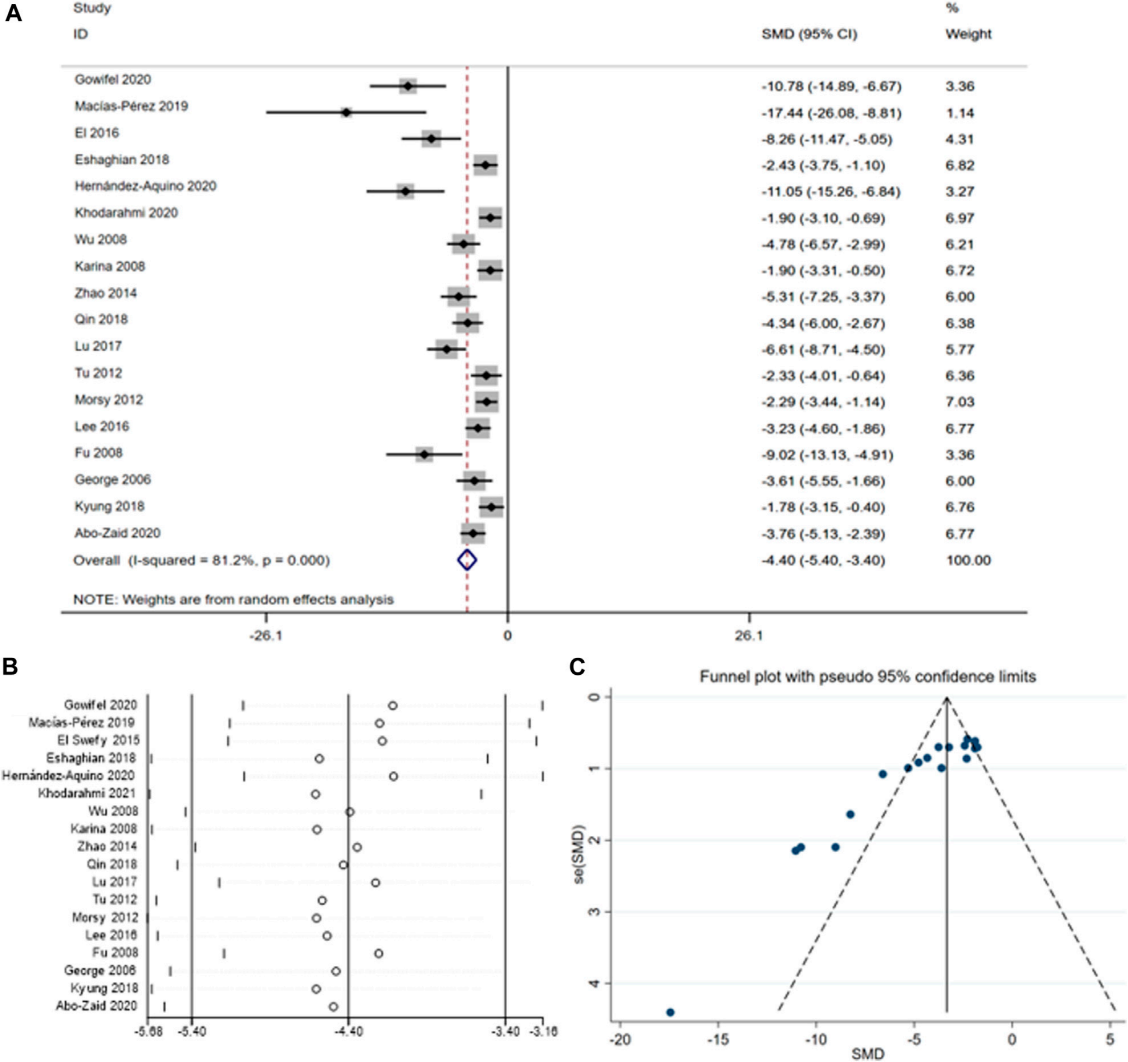
**(A)** Forest plot: antifibrosis effect of ALT; **(B)** sensitive analysis of ALT; **(C)** funnel plot of ALT.

To assess the impact of curcumin on ALT in fibrosis-model animals, we conducted a meta-analysis involving 18 studies encompassing a total of 296 animals (Gowifel et al., 2020; Macías-Pérez et al., 2019 El et al., 2016; Eshaghian et al., 2018; (Fu et al., 2008; George et al., 2006; Reyes-Gordillo et al., 2008; Wu et al., 2008; Morsy et al., 2012; Tu et al., 2012; Zhao et al., 2014; Lee et al., 2016; Lu et al., 2017; Kyung et al., 2018; Qin et al., 2018; Abo-Zaid, Shaheen, and Ismail, 2020; Hernández-Aquino et al., 2020; Khodarahmi et al., 2020). The summary analysis revealed a significant reduction in ALT levels [SMD = -4.40, 95% CI (−5.40, −3.40), *p* < 0.01, I^2^ = 81.2%, Figure 4A] with curcumin treatment. However, given the asymmetric funnel funnel plot (Figure 4B) and apparent heterogeneity (Figure 4C) among the different studies subgroup analyses were performed according to the species (Supplementary Figure S3) and the method for (Table 4).

**TABLE 4:** Subgroup analysis of ALT.

Subgroup	Number of paper	SMD	95% CI	*p*-value	Heterogeneity
Species					
Wistar rats	8	−4.35	−5.99, −2.72	*p* < 0.01	84.7
Sprague-Dawley rats	7	−3.92	−5.18, −2.65	*p* < 0.01	71.7
Method for molding					
CCI 4	11	−4.93	−6.22, −3.64	*p* < 0.01	79.1
BDL	4	−3.02	−4.72, −1.32	*p* < 0.01	78.5
NDMA	2	−2.56	−4.33, −0.79	*p* = 0.132	55.9

##### 3.4.1.3 ALP

**FIGURE 5:**
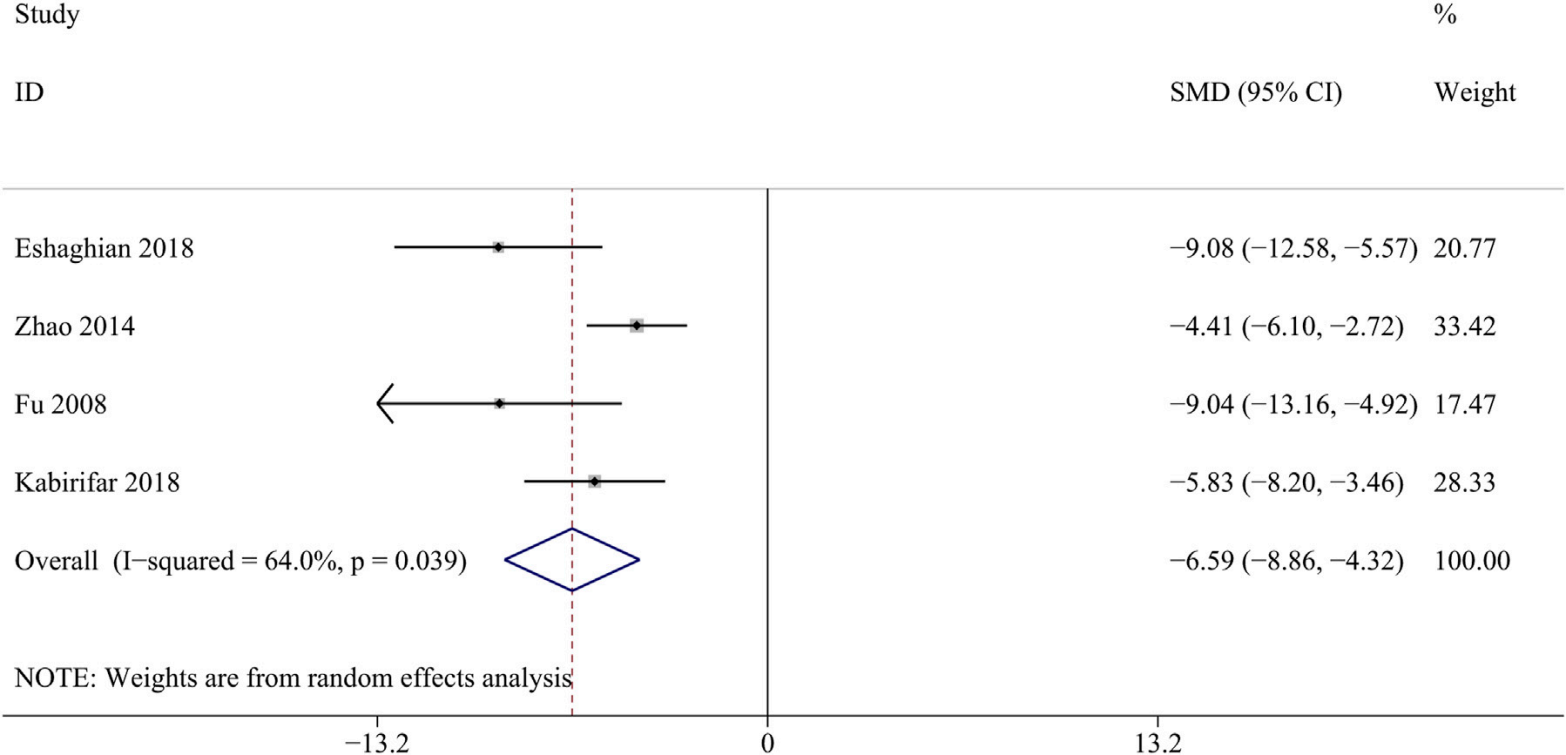
Forest plot: antifibrosis effect of curcumin on the ALP.

ALP is mainly produced in the liver, liver fibrosis results in liver damage and increased ALP synthesis and secretion (Liu et al., 2021). A total of 4 studies involving 296 animals were included in the analysis of the ALP levels (Eshaghian et al., 2018; Zhao et al., 2014; Fu et al., 2008; Kabirifar et al., 2018). Curcumin significantly reduced liver ALP levels compared with the model groups [SMD = − 6.59, 95% CI (−8.86,4.31), *p* < 0.05, I^2^ = 64.0%, Figure 5].

##### 3.4.1.4 ALB

**FIGURE 6:**
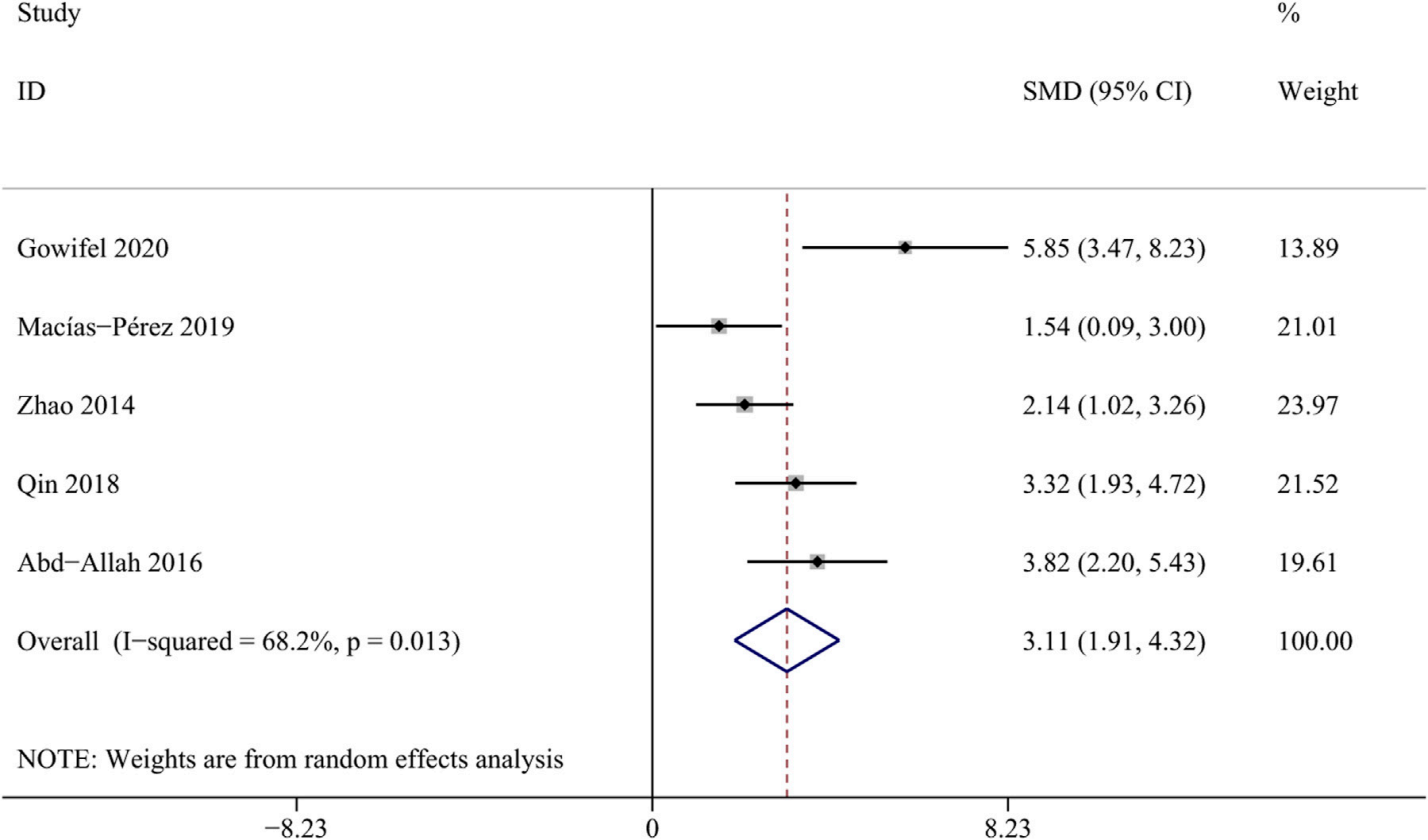
Forest plot: effects of curcumin on ALB.

Albumin is synthesized by hepatic parenchymal cells and reflects the function of the liver (Garcia-Martinez et al., 2013). A total of 5 studies involving 84 animals were included in this meta-analysis (Gowifel et al., 2020; Macías-Pérez et al., 2019; Zhao et al., 2014; Qin et al., 2018; Abd-Allah et al., 2016). The result revealed a significant increase in the albumin levels within the curcumin groups [SMD = 3.11, 95%CI (1.91,4.32), *p* = 0.013, I^2^ = 68.2%, Figure 6], suggesting that curcumin may contribute to enhancing liver synthetic function in the state of liver fibrosis.

##### 3.4.1.5 TBil

**FIGURE 7:**
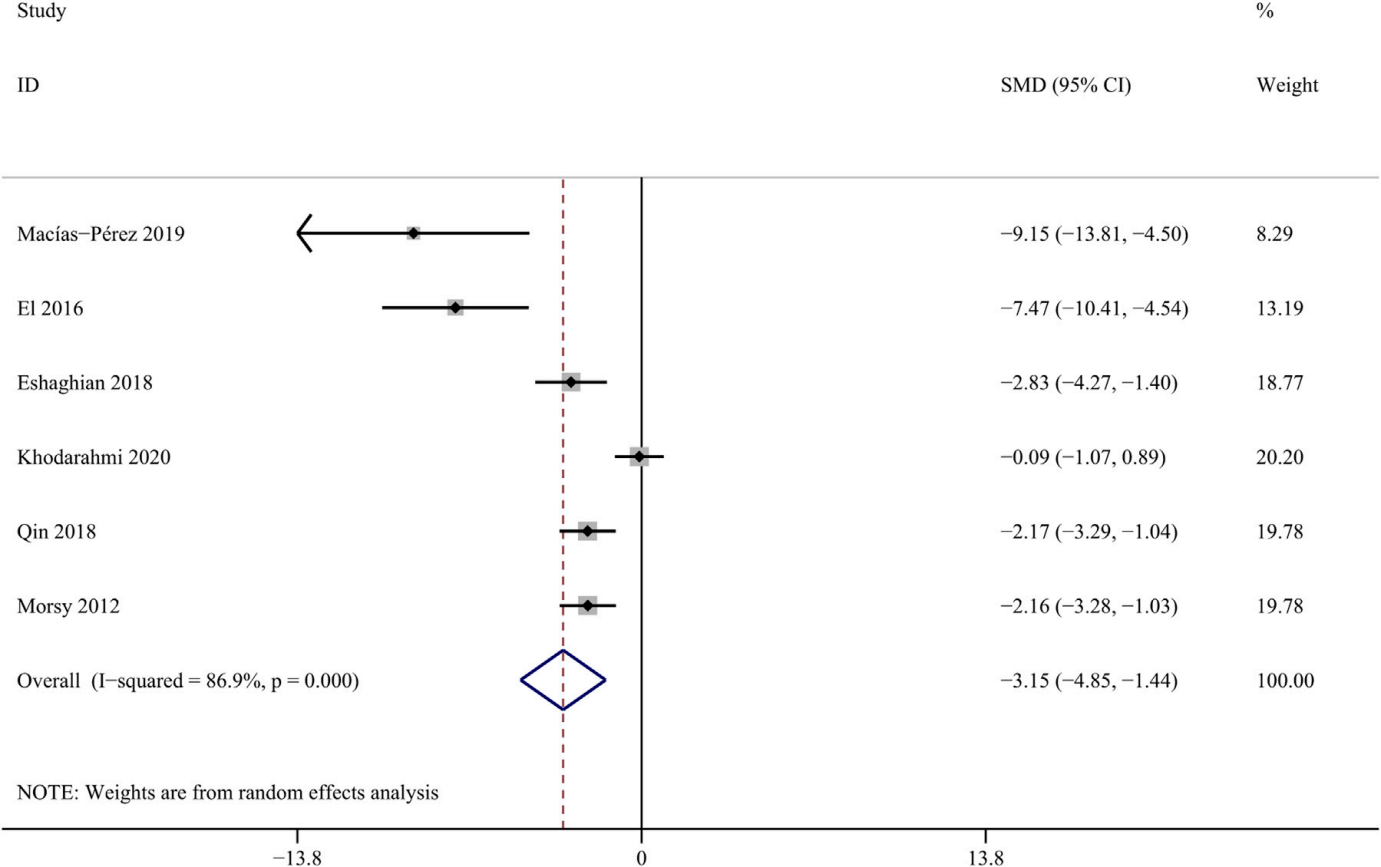
Forest plot: antifibrosis effect of curcumin on TBil.

Reduced excretory function of hepatocytes due to hepatic fibrosis, resulting in elevated serum total bilirubin (Tamber et al., 2023). Six studies involving 48 animals were included to assess the influence of curcumin on bilirubin level (Macías-Pérez et al., 2019; Zhao et al., 2014; Abd-Allah et al., 2016; Barta et al., 2015). The results indicate that curcumin can effectively reduce the concentration of TBil [SMD = − 3.15, 95% CI (−4.85,-1.44), *p* < 0.01, I^2^ = 86.9%, Figure 7].

#### 3.4.2 Collagen I

**FIGURE 8:**
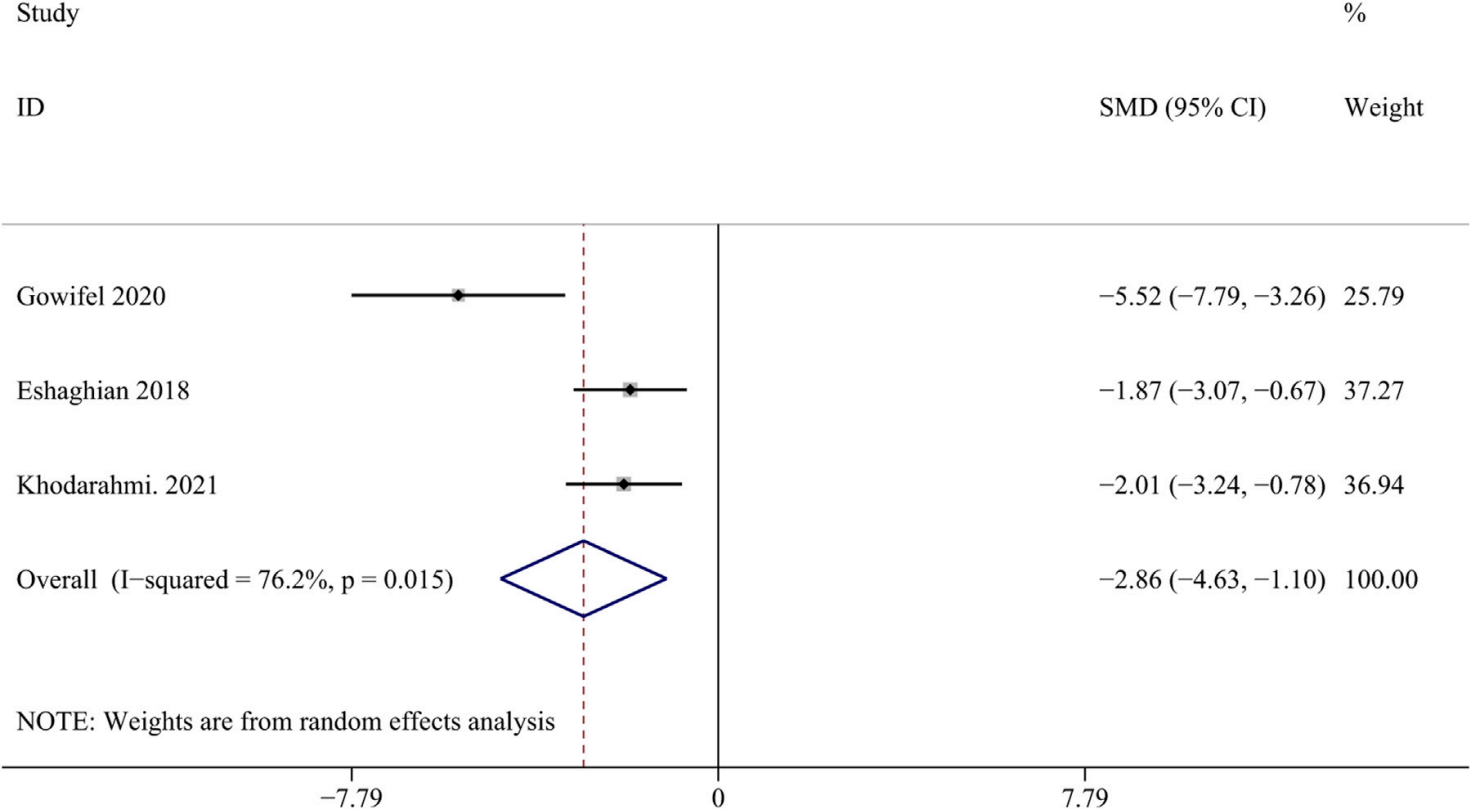
Forest plot: antifibrosis effect of curcumin on the collagen I.

Collagen I is an important biomarker for assessing scarring after liver injury (Mu et al., 2018). Three studies with 48 animals for collagen I were included in the meta-analysis (Gowifel et al., 2020; Hernández-Aquino et al., 2020; Eshaghian et al., 2018). The results demonstrated a decrease in the expression of collagen I with the application of curcumin [SMD = - 2.86, 95% CI (−4.63, −1.10), *p* = 0.015, I^2^ = 76.2%, Figure 8], indicating its potential anti-fibrotic effect.

#### 3.4.3 mRNA expression levels of α-SMA

**FIGURE 9:**
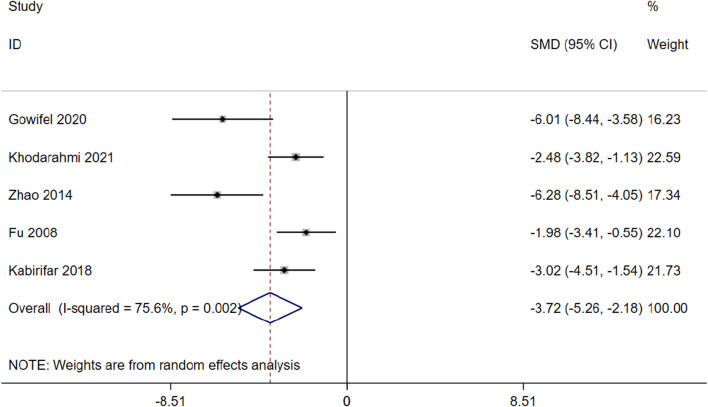
Forest plot: antifibrosis effect of curcumin on the mRNA of α-SMA.

α-SMA is a marker protein that indicates activation of HSCs and is indicative of liver fibrosis (Yuan et al., 2022). 5 studies involving 80 animals were included in the meta-analysis to assess the impact of curcumin on mRNA of α-SMA expression (Gowifel et al.; Hernández-Aquino et al., 2020; Zhao et al., 2014; Fu et al., 2008; Kabirifar et al., 2018). The results indicate that curcumin has the potential to enhance apoptosis and reduce mRNA of α-SMA expression [SMD = −3.72 (−5.26,-2.18), *p* < 0.01, I^2^ = 75.6%, Figure 9] in the context of hepatic fibrosis.

#### 3.4.4 Oxidative stress levels

Oxidative stress damage is one of factors inducing liver fibrosis. SOD, MDA, GSH and HYP are biomarkers of oxidative stress ((Ruart et al., 2019). To explore the relationship between curcumin treatment and oxidative stress levels, this meta-analysis incorporated three studies involving 52 animals focusing on serum MDA (Gowifel et al., 2020; El et al., 2016; Morsy et al., 2012), three studies involving 54 animals focusing on serum SOD (Gowifel et al., 2020; Wu et al., 2008; Abd-Allah et al., 2016), eight studies involving 134 animals focusing on serum GSH(Gowifel et al., 2020; (Hernández-Aquino et al., 2020; Reyes-Gordillo et al., 2008; James et al., 2018; Morsy et al., 2012; H.-Y. Lee et al., 2016; Fu et al., 2008; El et al., 2016); two studies involving 28 animals focusing on serum HYP((Kabirifar et al., 2018; Soto-Angona et al., 2020).

**FIGURE 10:**
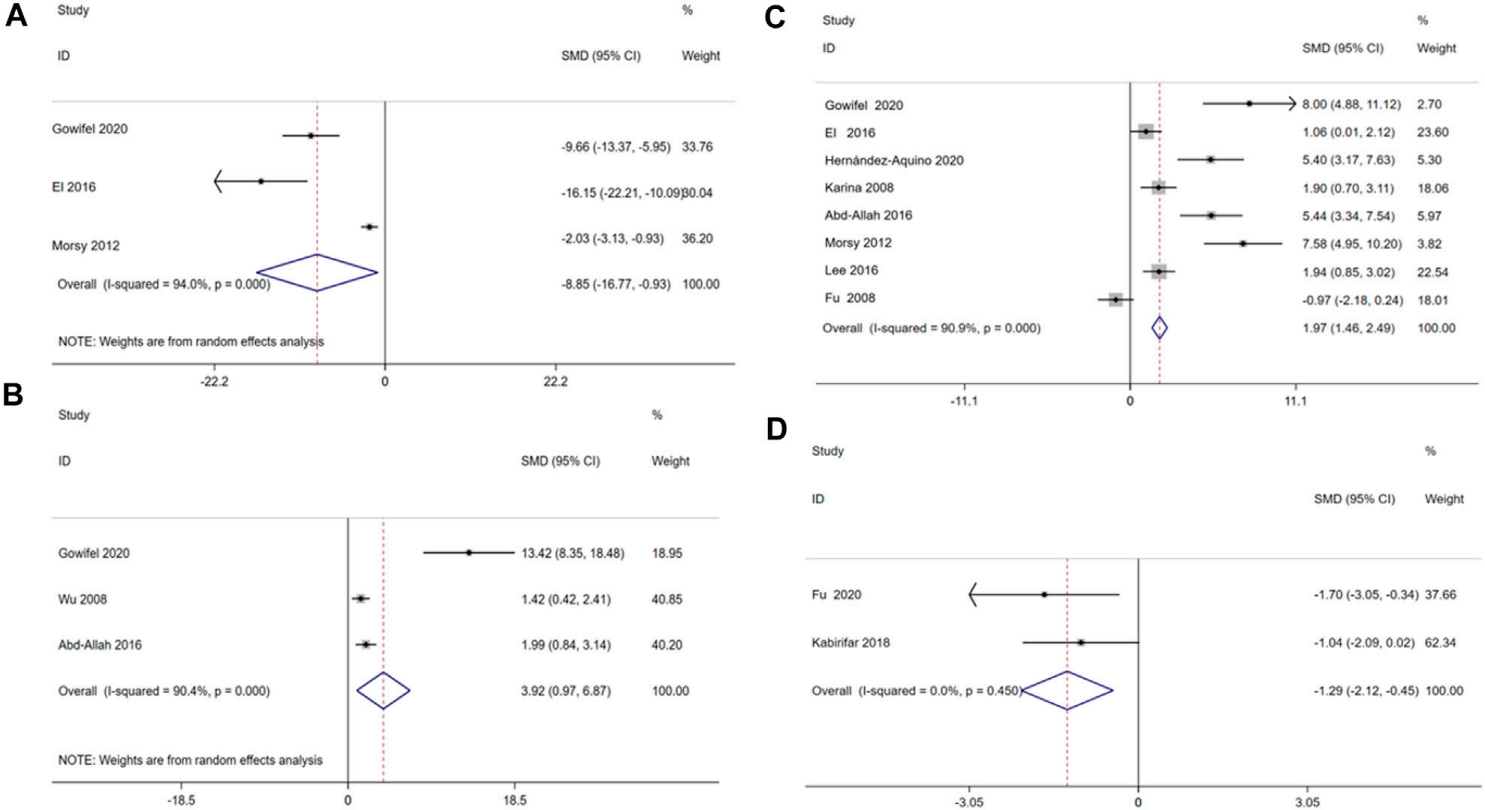
Forest plot: antifibrosis effect of curcumin on the Oxidative stress levels. **(A)** MDA; **(B)** SOD; **(C)** GSH; **(D)** HYP.

The result indicated that curcumin treatment reduced serum MDA [SMD = −8.85, 95% CI (−16.77, −0.93), *p* < 0.01, I^2^ = 94.0%, Figure 10A], increase serum SOD [SMD = 3.92, 95% CI (0.97,6.87), *p* < 0.01, I^2^ = 90.4%, Figure 10B] and serum GSH [SMD = 1.97, 95% CI (1.46, 2.49), *p* < 0.01, I^2^ = 90.9%, Figure 10C] in a significant manner, and reduced serum HYP [SMD = −1.29, 95% CI (−2.12, −0.45), *p* = 0.450, I^2^ = 0.0%, Figure 10D].

#### 3.4.5 Regulation of pathway factors

##### 3.4.5.1 Levels of TGF-β mRNA

**FIGURE 11:**
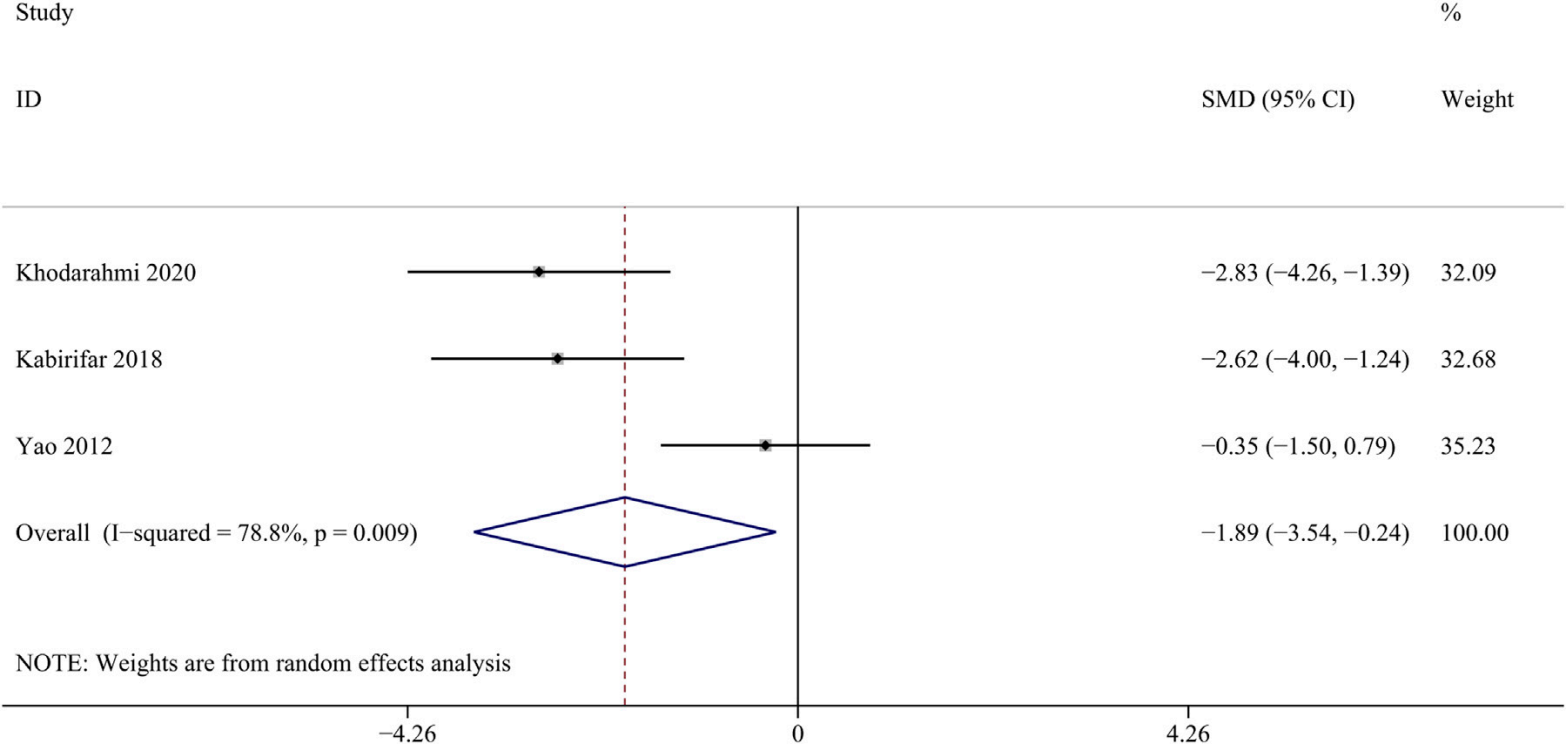
Forest plot: antifibrosis effect of curcumin on the mRNA expression levels of TGF-β

TGF-β participates in regulating cell growth, proliferation, and differentiation, and is an important regulatory factor in the process of liver fibrosis (Seki et al., 2007). Three studies with 28 animals for mRNA of TGF-β levels were included in the meta-analysis (Khodarahmi et al., 2020; Kabirifar et al., 2018; Yao et al., 2012). The results demonstrated a decrease in the expression of mRNA of TGF-β levels with the treatment of curcumin [SMD = − 1.89, 95% CI (−3.54, −0.24), *p* < 0.01, I^2^ = 78.8%, Figure 11].

##### 3.4.5.2 Levels of NF-κb mRNA

**FIGURE 12:**
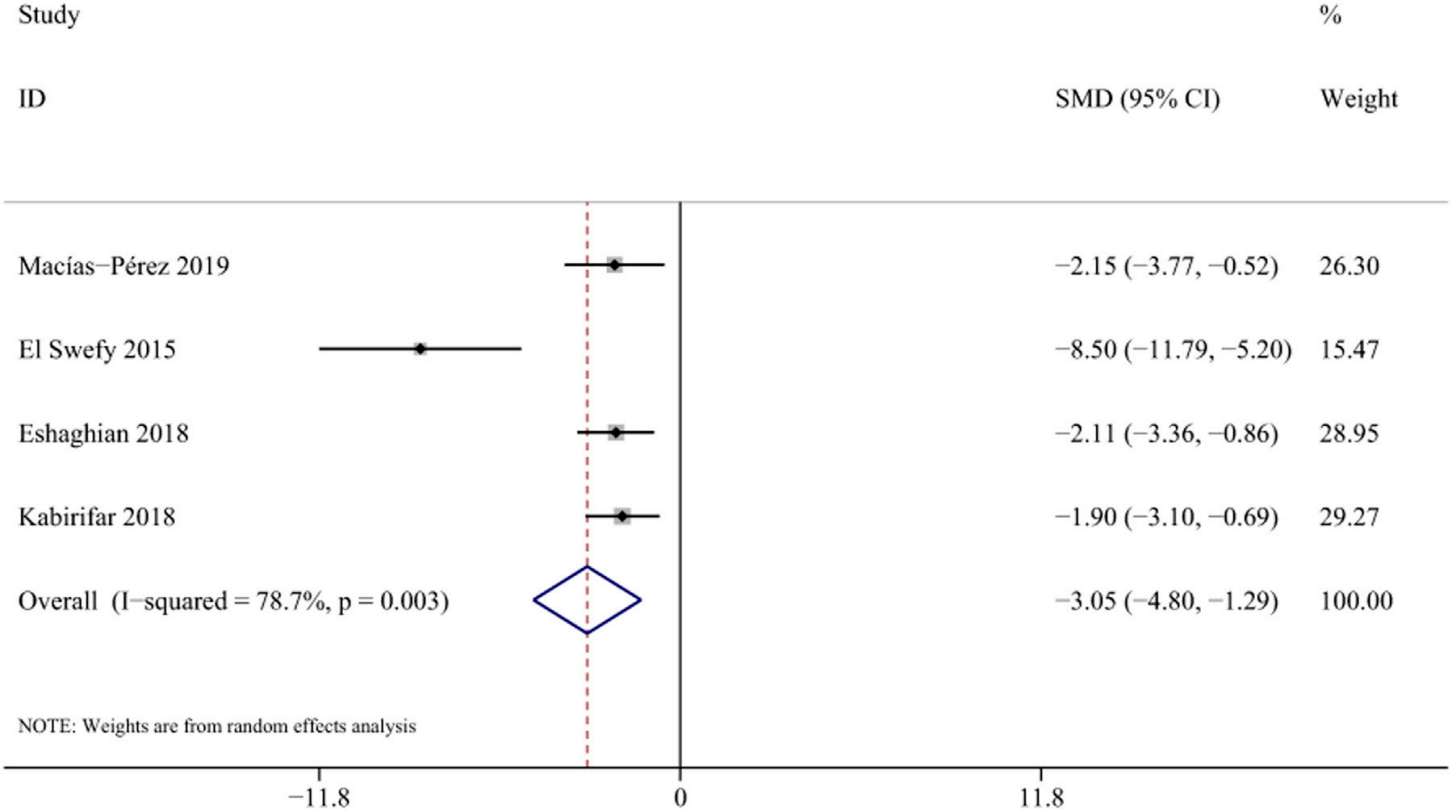
Forest plot: antifibrosis effect of curcumin on the mRNA expression levels of NF-κb.

NF-κB activation is closely associated with inflammatory responses and can promote the activation of HSCs and proliferation of fibroblasts. (Luedde and Schwabe, 2011). Four studies with 58 animals for mRNA of NF-κb levels were included in the meta-analysis (Macías-Pérez et al., 2019; El et al., 2016; Eshaghian et al., 2018; Kabirifar et al., 2018). The results demonstrated a decrease in the expression of mRNA of NF-κb levels with the treatment of curcumin [SMD = −3.05, 95% CI (−4.80,-1.29), *p* < 0.01, I^2^ = 78.7%, Figure 12].

#### 3.4.6 Triglyceride

**FIGURE 13:**
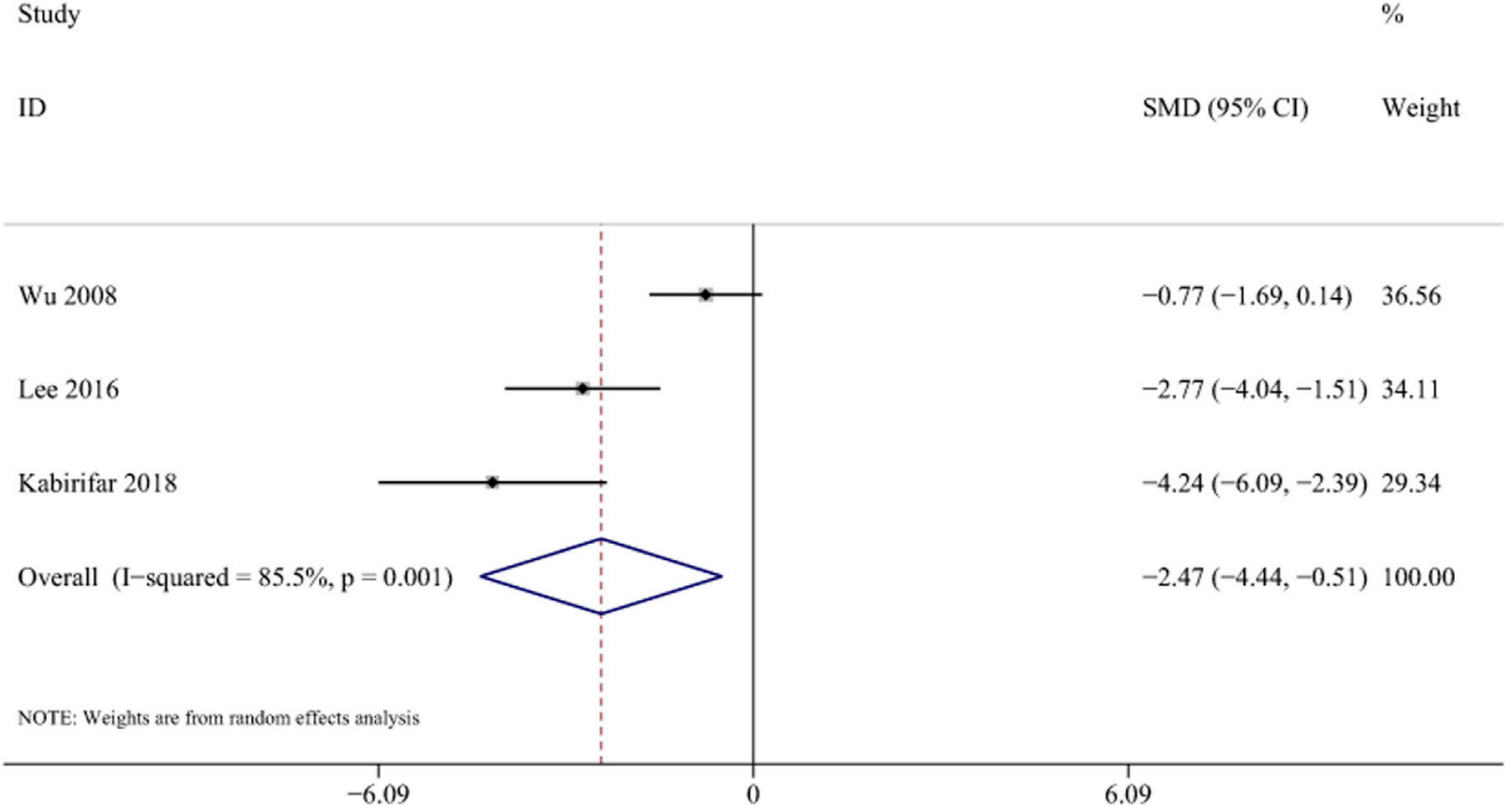
Forest plot: antifibrosis effect of curcumin on TG.

Three studies with 56 animals for mRNA of TG levels were included in the meta-analysis (Wu et al., 2008; Lee et al., 2016; Kabirifar et al., 2018). The results demonstrated a decrease in the expression of TG levels with the treatment of curcumin [SMD = −2.47, 95% CI (−4.44,-0.51), *p* < 0.01, I^2^ = 85.5%, Figure 13].

#### 3.4.7 Laminin

**FIGURE 14:**
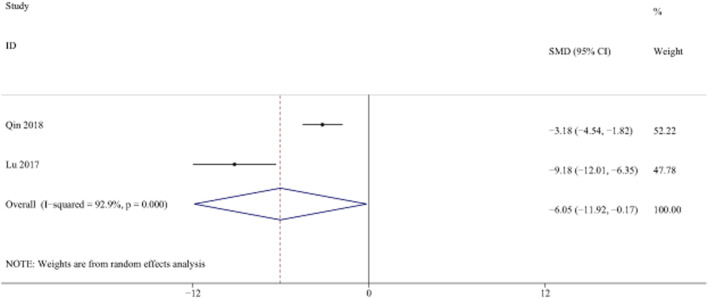
Forest plot: antifibrosis effect of curcumin on LN.

LN is a key component of ECM, which is a marker of liver fibrosis. (Plevris et al., 2018). Two studies with 44 animals for LN levels were included in the meta-analysis (El et al., 2016; Lu et al., 2017; Qin et al., 2018). The results demonstrated a decrease in the expression of LN levels with the treatment of curcumin [SMD = −6.05, 95% CI (−11.92, −0.17), *p* < 0.01, I^2^ = 92.9%, Figure 14].

#### 3.4.8 Degree of fibrosis

**FIGURE 15:**
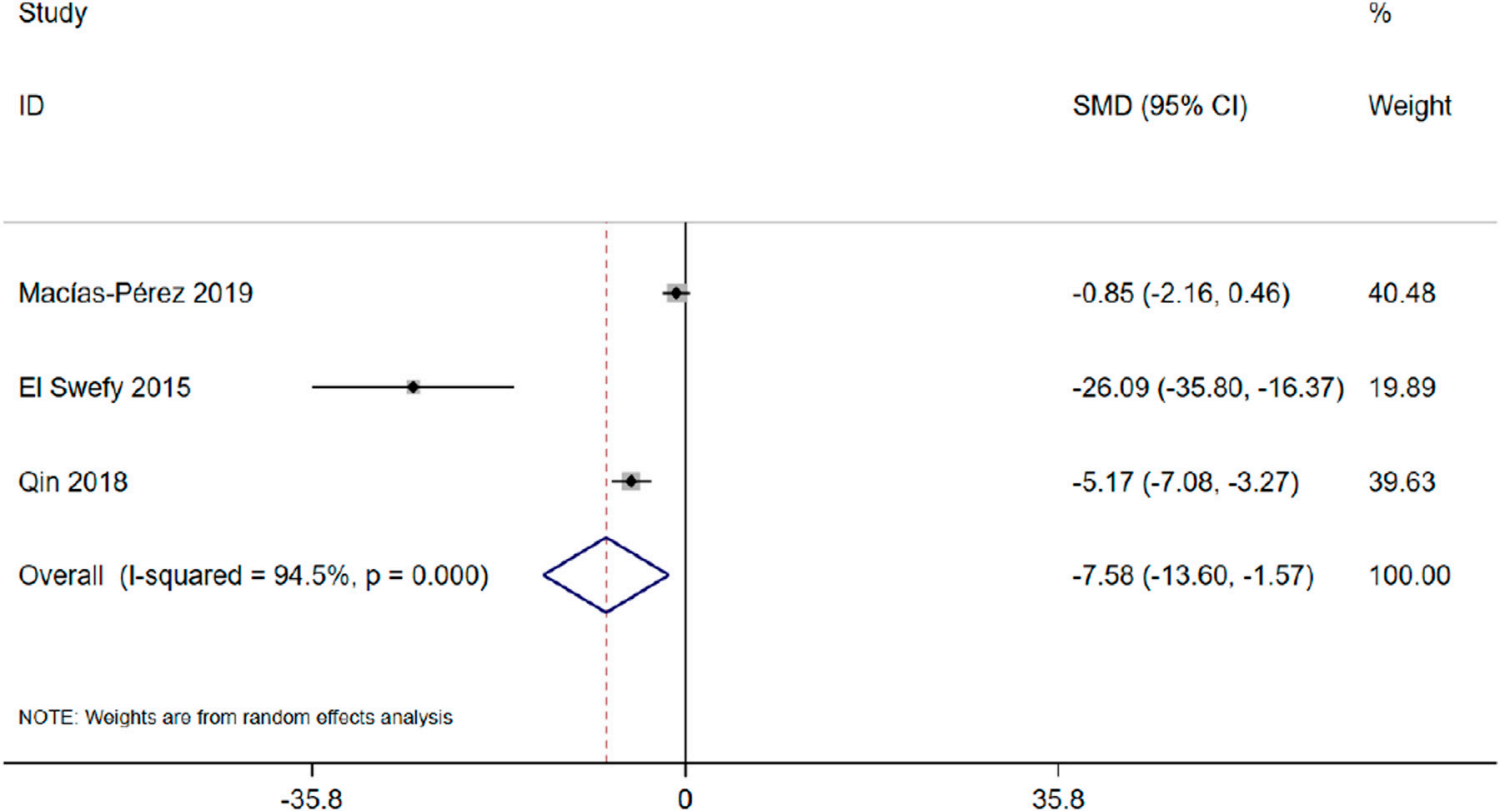
Forest plot: antifibrosis effect of curcumin on the degree of fibrosis.

Three studies with 46 animals for the degree of fibrosis were included in the meta-analysis (Macías-Pérez et al., 2019; Qin et al., 2018). The results demonstrated a decrease in the degree of fibrosis with the treatment of curcumin [SMD = −7.58, 95% CI (−13.60, −1.57), *p* < 0.01, I^2^ = 94.5%, Figure 15].

#### 3.4.9 Liver histopathology

Pathological examinations, including hematoxylin and eosin (H&E) staining, Masson trichromatic (MT) staining, and Sirius red staining, were performed in 23 included papers. Specifically, H&E staining was conducted in 21 studies, Sirius red staining in 6 studies, and MT staining in 10 studies. Liver histological examinations using H&E and Compared with the model groups, curcumin groups showed a significant reduction in inflammatory cell infiltration and interstitial collagen fiber deposition. Moreover, it promoted a delay in hepatocyte necrosis and apoptosis, protecting the normal liver tissue structure. MT staining demonstrated a significant reduction in collagen production in the liver, while Sirius red staining indicated that curcumin could reduce collagen fiber generation compared to the model group.

## 4 Discussion

### 4.1 Summary of evidence

Curcumin was evaluated in this study for its efficacy in treating experimental liver fibrosis. In our meta-analysis, consisting of 24 articles included 440 animals, and the overall quality of the selected literature was medium to high. A comprehensive analysis revealed that curcumin intervention not only mitigates liver fibrosis but also improves liver function, while concurrently modulating inflammatory responses and antioxidant capacity in animal models. This result provided a strong basis for further large-scale animal studies as well as clinical trials in humans in the future.

### 4.2 Molecular mechanisms

**FIGURE 16:**
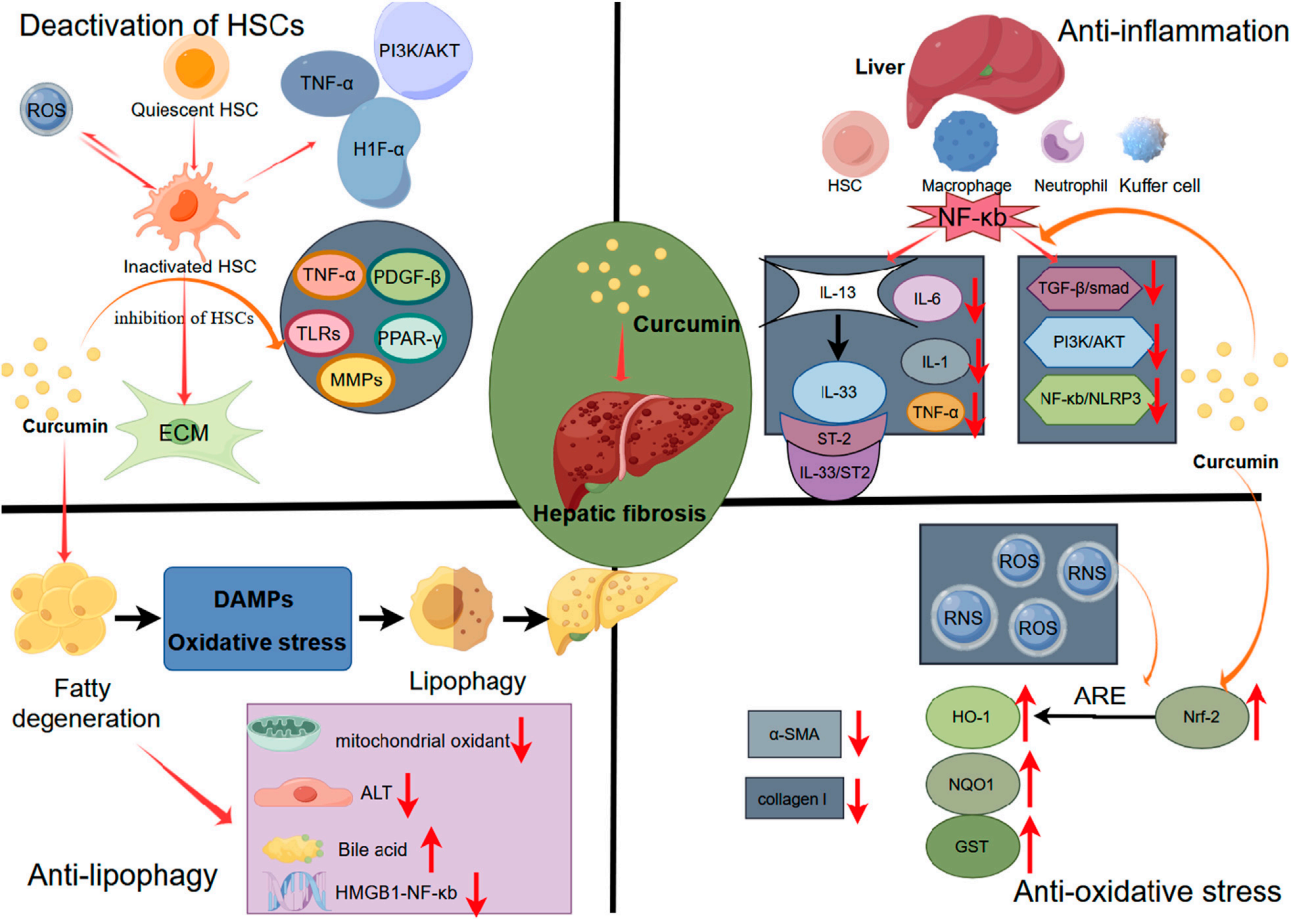
Potential mechanism of curcumin to improve hepatic fibrosis.

The pathological process of liver fibrosis encompasses activation of HSCs, inflammation, oxidative stress, etc., (Yang et al., 2023). With its hepatoprotective and anti-fibrotic effects, curcumin exerts its mechanisms through antioxidative and anti-inflammatory actions, inhibition of hepatic stellate cell activation, and the blockade of receptors and signaling pathways (Farzaei et al., 2018). We delved into the molecular mechanisms underlying these processes (Figure 16).

#### 4.2.1 Curcumin improves liver fibrosis by inhibiting HSC activation

HSCs are nonparenchymal cells in the liver, constituting 5%–10% of the total liver cell population. HSCs usually remain in a quiescent state, displaying low proliferative activity and limited collagen synthesis (Higashi, Friedman, and Hoshida, 2017). However, under liver stress, HSCs can undergo activation, transitioning from a ‘static’ to an ‘active’ phenotype. This activation enhances their collagen synthesis capability, leading to the secretion of ECM and pro-inflammatory mediators (Heymann and Tacke, 2016). This process contributes to liver fibrosis, serving as a key driver in liver injury (Kamm and McCommis, 2022).

In the progression of HSC-mediated liver fibrosis, various cytokines and cellular pathways interplay, creating an intricate network of interactions, including TGF-β, PI3K/AKT, MAPK, HIF-1α, Wnt signaling, and downstream pathways ((Xiang et al., 2018) (J. Wang et al., 2018). In addition, HSCs can generate reactive oxygen species and nitrogen compounds (ROS and RNS), which can induce HSC activation, proliferation, and apoptosis. These by-products result from metabolic events associated with HSC activation (Khomich, Ivanov, and Bartosch, 2019).

Numerous studies demonstrated that curcumin effectively targets the inhibition of HSC activation (Shu et al., 2023). intervened with curcumin in the HSC line LX-2 and found that curcumin could inhibit activity and promote apoptosis in LX-2 cells by suppressing autophagy through activation of the PI3K/Akt/mTOR signaling pathway (Shu et al., 2023). Lian et al. discovered that curcumin inhibits glycolysis and regulates metabolism in HSCs by modulating hedgehog signaling (Lian et al., 2015). Qin et al., in their intervention with curcumin on HSC-T6 cells, found that curcumin could protect against activation and migration of hepatic stellate cells by inhibiting the CXCL12/CXCR4 biological axis in liver fibrosis (Qin et al., 2018).

#### 4.2.2 Curcumin prevents liver fibrosis by modulating the inflammatory response

Decades of chronic inflammation can lead to structural damage in the liver, where normal tissue is gradually replaced by scar tissue (Czaja, 2014). This process results in impaired organ function, altered hepatic blood flow, and the development of liver fibrosis. Inflammatory cells found in the liver, including Kupffer cells, macrophages, neutrophils, and hepatic stellate cells, play a crucial role in this progression (Koyama and Brenner, 2017). Under sustained stimulation, these cells release pro-inflammatory mediators such as NF-κB, IL-6, IL-22, or TGF-β. These mediators, in turn, facilitate the recruitment of T cells and neutrophils, while also stimulating the fibrotic activity of HSCs(Hammerich and Tacke, 2023).

In the present study, rat and human fibrotic livers expressed elevated levels of both IL-33 and ST2, compared to healthy livers (Moussion, Ortega, and Girard, 2008). The IL-33 and ST-2 axis contribute to signaling via the ST 2L receptor, which contains a membrane binding domain, an extracellular segment containing three interconnected motifs similar to immunoglobulins, and a cytoplasmic Toll/IL-1 receptor domain.

(Pascual-Figal and Januzzi, 2015; Marvie et al., 2010). In chronic injury, IL-33 serves as a factor contributing to liver fibrosis, and ST2 acts as an important biomarker for liver fibrosis (Mchedlidze et al., 2013; Oztas et al., 2015). Furthermore, their expression demonstrated a significant rise in tandem with the severity of fibrosis (Gao et al., 2016). Additionally, it has been discovered that IL-13 primarily triggers the pro-fibrotic impact of IL-33. By stimulating TGF-β signaling via IL-4 Rα and signal transducers and transcriptional activator 6 (STAT 6) in HSCs, IL-13 can facilitate liver fibrosis (Liang et al., 2019).

The NF-κB signaling plays crucial role in regulating immune and inflammatory responses (Zhang et al., 2019). NF-κB activation triggers the release of inflammatory cytokines and chemokines, such as TGF-β1, TNF-α, IL-1, IL-6, and IFN-γ(Hayden and Ghosh, 2008). Moreover, it participates in the activation of other pathways, such as the NF-κB/IκBα signaling pathway and the downstream NF-κB/NLRP3 signaling pathway (Pariente-Pérez, et al., 2020), thereby exacerbating liver inflammation and fibrosis. NF-κB governs liver fibrosis through three key mechanisms: (A) overseeing liver cell damage, acting as the primary initiator of the fibrotic response; (B) adjusting inflammatory signals initiated in liver macrophages and other inflammatory cells; and (C) impacting the fibrotic response in HSC(Luedde and Schwabe, 2011). Curcumin has been documented to inhibit NF-κB phosphorylation and degradation, decrease p65 expression, hinder the activation of the NF-κB signaling pathway, and alleviate hepatic inflammation (Barta et al., 2015).

Wu et al. investigated the effect of curcumin on CCI4-induced liver fibrosis in mice and demonstrated that curcumin inhibits NF-κB and IL-6 expression while enhancing the expression of the anti-inflammatory factor IL-10, thereby exerting an anti-fibrotic role (Wu et al., 2008). Similarly, Hernández-Aquino et al. treated CCI4-induced Wistar rats with curcumin and observed a reduction in liver fibrosis. They also noted that curcumin restored protein levels of NF-κB, IL-1, IL-10, TGF-β, CTGF, Col-I, and Smad7 (Hernández-Aquino et al., 2020). Furthermore, Wang et al. found that curcumin treatment increased the ratio of Nrf-2/NF-κB mRNA and its protein expression in liver inflammatory cells, protecting the liver and reversing the process of cirrhosis (Wang et al., 2018).

#### 4.2.3 Curcumin inhibits liver fibrosis by regulating fat metabolism

The development of liver fibrosis is closely linked to disturbances in fat metabolism. According to the “second blow” theory, the accumulation of free fatty acids (FFA), coupled with inflammation or insulin resistance, can give rise to pathological manifestations, including lipid accumulation, hepatocyte ballooning, lobular inflammation, and fibrosis (Soto-Angona et al., 2020). From NAFLD to NASH, oxidative stress, damage-associated molecular patterns (DAMPs), and other byproducts of cellular metabolic disorders, induced by an excessive lipid burden, continuously trigger the process of lipophagy. Lipophagy, a lysosomal-mediated lipid metabolism process, assists the liver in eliminating metabolic waste. The overactivation of HSCs induced by lipophagy promotes ECM deposition, speeding up the advancement of liver fibrosis (Filali-Mouncef et al., 2022).

Research indicates that curcumin reduces serum cholesterol levels by increasing hepatic LDL receptor expression, inhibiting LDL oxidation, and enhancing bile acid secretion and faecal cholesterol excretion (Zhang et al., 2019). Curcumin also suppresses genes involved in cholesterol biosynthesis, proecting against liver injury and fibrogenesis in animal models (Fu et al., 2008; Peschel et al., 2007). Using autophagy inhibitors in NASH treatment prevents *in vitro* HSC activation and reduces lipid droplet degradation (Thoen et al., 2011), suggesting autophagy as a potential target for NASH and fibrosis treatment. Afrin et al. demonstrated that curcumin mitigates liver damage and inhibits the progression of NASH and fibrosis in model neonatal C57BL/6J male mice induced by streptozotocin by inhibiting HMGB1-NF-κB translocation (Afrin et al., 2017). Furthermore, supplementing curcumin to hyperadipose-induced NASH in experimental rabbits reduced NASH grade and aminotransferase activity, while increasing mitochondrial antioxidant levels (Ramirez-Tortosa et al., 2009).

#### 4.2.4 Curcumin inhibits liver fibrosis by regulating oxidative stress

ROS is produced by oxidative stress in the liver for various reasons, such as chronic viral infection, drug hepatotoxicity and alcohol damage, which induces hepatocyte necrosis and subsequent liver function damage, leading to excessive deposition of ECM and diffuse liver fibrosis (Parola and Pinzani, 2019). Cellular antioxidant mechanisms are triggered in response to oxidative stress injury. ROS overproduction activates the transcription factor nuclear factor E2-related factor 2 (Nrf2). Through antioxidant response elements (ARE), Nrf2 can activate the expression of downstream antioxidant protection genes such as heme oxygenase 1 (HO-1), generating antioxidant enzymes like HO-1, NADH quinone oxidoreductase (NQO1), and glutathione S-transferase (GST), thereby protecting liver cells from oxidative damage (W. Tang, 2014). Prior research has shown that the overexpression of Nrf2, along with downstream antioxidant factors like NQO1 and HO-1, can diminish the expression of α-SMA, collagen I and collagen III in rats with liver fibrosis, exerting an anti-fibrotic effect (Ge et al., 2022).

Excessive levels of ROS can damage cellular lipids and proteins, leading to hepatocyte necrosis and apoptosis. Additionally, ROS promote the production of pro-inflammatory and pro-fibrogenic factors by activating HSCs, Kupffer cells, and other inflammatory cells (Luedde and Schwabe, 2011; Zhang et al., 2013). Studies have demonstrated that ROS signaling also regulates the expression and activity of the transcription factor NF-κB (Luedde and Schwabe, 2011). Inhibition of NF-κB activity has been shown to protect against hepatic fibrosis *in vivo* (Lee et al., 2020; Rodríguez et al., 2021). Kong et al. showed that curcumin inhibits ROS levels and oxidative stress in hepatocytes by activating PPAR-α and regulating upstream signaling pathways of autophagy, including AMPK and PI3K/AKT/mTOR (Kong et al., 2020). These effects contribute to the anti-liver fibrosis properties of curcumin.

### 4.3 Limitations

We need to acknowledge several potential limitations in our meta-analysis. Firstly, we limited the inclusion of literature to English, which may introduce selection bias by excluding studies published in other languages. Secondly, some literature overlooked the implementation of randomization and blinding methods, possibly compromising the authenticity of the results. Moreover, the meta-analysis revealed high heterogeneity in AST, ALT, ALP, ALB, and other indicators. Subgroup analysis based on animal species and modeling methods was conducted, but the heterogeneity remained largely unchanged. Through comprehensive literature review identified high heterogeneity as a common issue in experimental meta-analyses. The conduct of experiments may contribute to selection bias, given that positive results are more likely to be reported (Feng et al., 2022). These factors limit the generalizability of our results to clinical settings. Therefore, future studies should enhance methodological rigor, including strict adherence to randomization and blinding methods, provision of accurate experimental data, and appropriate expansion of sample sizes.

Curcumin, a hydrophobic polyphenol extracted from the rhizome of Curcuma longa, is widely utilized in the treatment of cardiovascular diseases, liver diseases, and tumours (Xu et al., 2020; Jabczyk et al., 2021; Pourbagher-Shahri et al., 2021). Despite curcumin exhibits challenges such as low aqueous solubility, poor stability in body fluids, rapid metabolism, and reduced absorption in the gastrointestinal tract, clinical and basic studies have confirmed its remarkable pharmacological efficacy in treating liver diseases like NAFLD, liver fibrosis, and liver cancer (Nelson et al., 2017; Farzaei et al., 2018); (E. S. Lee et al., 2020) The ongoing enhancement of nano-formulations of curcumin holds promise for expanding its clinical applications (Y. Chen et al., 2020).

## 5 Conclusion

Our meta-analysis showcased curcumin’s effectiveness in preclinical liver fibrosis studies. The potential protective mechanisms observed in animals encompass liver protection, collagen production inhibition, oxidative stress reduction, and inflammatory response regulation. Despite these promising findings, the predominance of animal studies underscores the necessity for clinical trials to validate curcumin’s clinical efficacy and precisely elucidate its mechanisms in liver fibrosis treatment.

## Figures and Tables

**Table:** 

**AKT**	protein kinase B
**ALB**	albumin
**ALD**	alcoholic liver disease
**ALP**	alkaline phosphatase
**ALT**	alanine aminotransferase
**ARE**	antioxidant response elements
**AST**	aspartate aminotransferase
**α-SMA**	alpha-smooth muscle actin
**BDL**	bile duct ligation
**CI**	confidence interval
**DAMPs**	damage-associated molecular patterns
**ECM**	extracellular matrix
**FFA**	free fatty acids
**GSH**	Glutathione
**HBV**	hepatitis B virus
**HCV**	hepatitis C virus
**HE**	hematoxylin and eosin
**HIF-1α**	hypoxia-inducible factor-1α
**HMGB1**	high-mobility group protein
**HO-1**	heme oxygenase 1
**HSC**	hepatic stellate cells
**HYP**	Hydroxyproline
**IFN-γ**	interferon-γ
**IκBα**	Inhibitor of NF-κB
**IL-1**	interleukin-1
**IL-6**	interleukin-6
**IL-13**	interleukin-13
**IL-22**	interleukin-22
**IL-33**	interleukin-33
**LDL**	low-density lipoprotein cholesterol
**LN**	laminin
**MAPK**	mitogen-activated protein kinase
**MDA**	malondialdehyde
**MMPs**	matrix metalloproteinase
**MT**	Masson trichromatic
**NAFLD**	non-alcoholic fatty liver disease
**NDMA**	N-Nitrosodimethylamine
**NF-κb**	nuclear factor κB
**NLRP3**	NOD-like receptor thermal protein domain associated protein 3
**NQO1**	NADH quinone oxidoreductase
**Nrf2**	nuclear factor E2-related factor
**PDGF-β**	platelet-derived growth factor receptor-β
**PI3K**	phosphoinositide 3-kinase
**RNS**	reactive nitrogen species
**ROS**	reactive oxygen species
**PPAR-γ**	peroxisome proliferator-activated receptor
**Smad**	*Drosophila* mothers against decapentaplegic protein
**SD**	standard deviation
**SEM**	Standard Error of the Mean
**SMD**	standard mean difference
**SOD**	superoxide dismutase
**STAT 6**	transcriptional activator 6
**ST-2**	growth stimulation expressed gene 2
**TAA**	Thioacetamide
**Tbil**	total bilirubin
**TGF-β**	transforming growth factor-beta
**TLRs**	Toll-like receptors
**TP**	total protein
**TG**	triglyceride

## Data Availability

The original contributions presented in the study are included in the article/Supplementary Material, further inquiries can be directed to the corresponding author.
