# Supplementary material for: A role for curcumin in preventing liver fibrosis in animals: a systematic review and meta-analysis
Source: Front Pharmacol. 2024 May 24;15:1396834. doi: 10.3389/fphar.2024.1396834 (PMC11157132; doi:10.3389/fphar.2024.1396834)
Supplement: Supplementary file 1 [file DataSheet1.pdf]

Supplementary materials

Supplementary 1

Literature search strategy for curcumin in the treatment of Hepatic fibrosis

1.1 Pubmed search formation

#1("liver cirrhosis"[MeSH Terms]

#2 ("liver"[All Fields] AND "cirrhosis"[All Fields]) OR "liver cirrhosis"[All Fields] OR "cirrhosis liver"[All Fields] OR ("hepatic"[All Fields] AND "cirrhosis"[All Fields]) OR "hepatic cirrhosis"[All Fields] OR "cirrhosis hepatic"[All Fields] OR ("fibrosis"[All Fields] AND "liver"[All Fields]) OR "fibrosis liver"[All Fields]) OR "liver fibrosis"[All Fields] OR ("hepatic"[All Fields] AND "fibrosis"[All Fields]) OR "hepatic fibrosis"[All Fields] OR "fibrosis hepatic"[All Fields]

#3 #1 OR #2

#4 ("curcumin "[MeSH Terms])

#5 "curcumin"[MeSH Terms] OR ("curcumin"[MeSH Terms] OR "curcumin"[All Fields] OR ("turmeric"[All Fields] AND "yellow"[All Fields]) OR "turmeric yellow"[All Fields] OR ("curcumin"[MeSH Terms] OR "curcumin"[All Fields] OR ("yellow"[All Fields] AND "turmeric"[All Fields]) OR "yellow turmeric"[All Fields]) OR ("curcumin"[MeSH Terms] OR "curcumin"[All Fields] OR ("curcumin"[All Fields] AND "phytosome"[All Fields]) OR "curcumin phytosome"[All Fields]) OR ("curcumin"[MeSH Terms] OR "curcumin"[All Fields] OR ("phytosome"[All Fields] AND "curcumin"[All Fields])) OR ("curcumin"[MeSH Terms] OR "curcumin"[All Fields] OR "diferuloylmethane"[All Fields]) OR ("curcumin"[MeSH Terms] OR "curcumin"[All Fields]))

#6 #4 OR #5

#7 #3 AND #6

1.2 Web of science search formation

#1 TS=(liver cirrhosis OR Hepatic Cirrhosis OR Cirrhosis, Hepatic OR Cirrhosis, Liver OR Fibrosis, Liver OR Liver Fibrosis) 105626

#2 TS=(Curcumin OR 1,6-Heptadiene-3,5-dione, 1,7-bis(4-hydroxy-3-methoxyphenyl)-, (E,E) OR Turmeric Yellow OR Yellow, Turmeric OR Curcumin Phytosome OR Phytosome, Curcumin OR Diferuloylmethane OR Mervia) 30440

#3 #1 AND #2

1.3 EMBASE Search formation

#1 'liver cirrhosis'/exp OR 'liver cirrhosis' 218393

#2 'liver cirrhosis':ab,kw,ti OR 'Hepatic Cirrhosis':ab,kw,ti OR 'Cirrhosis, Hepatic': ab,kw,ti OR 'Cirrhosis, Liver':ab,kw,ti OR 'Fibrosis, Liver':ab,kw,ti OR 'Liver Fibrosis':ab,kw,ti

#3 #1 or #2

#4 'curcumin'/exp OR 'curcumin'

#5 'Curcumin':ab,kw,ti OR '1,6-Heptadiene-3,5-dione, 1,7-bis(4-hydroxy-3-methoxyphenyl)-, (E,E)-':ab,kw,ti OR ' Turmeric Yellow':ab,kw,ti OR 'Yellow, Turmeric':ab,kw,ti OR 'Curcumin Phytosome':ab,kw,ti OR 'Phytosome, Curcumin':ab,kw,ti OR 'Diferuloylmethane':ab,kw,ti OR 'Mervia':ab,kw,ti 26574

#6 #4 OR #5

#7 #3 AND #6

#8 #3 AND #6 AND [animals]/lim

1.4 Cochrane search formation

#1 MeSH descriptor: [Liver Cirrhosis] explode all trees

#2 (liver cirhosis) OR (Hepatic Cirrhosis) OR (Cirrhosis, Hepatic) OR (Cirrhosis, Liver) OR (Fibrosis, Liver):ti,ab,kw

#3 (Liver Fibrosis)

#4 #1 OR #2 OR #3

#5 MeSH descriptor: [Curcumin] explode all trees

#6 (Curcumin) OR (Phytosome, Curcumin) OR (Turmeric Yellow) OR (Yellow, Turmeric) OR (Curcumin Phytosome)

#7 (Diferulovlmethane) OR (Mervia)

#8 #5 OR #6 OR #7

#9 #4 AND #8

Supplementary 2

Table 1 Risk of bias summary

(First author et al., year)	A	B	C	D	E	F	G	H	I	J	Score
Gowifel, et al., 2020	+	+	-	+	-	-	?	+	+	+	6
Macías-Pérez et al., 2019	?	+	-	+	-	-	?	+	+	+	5
El et al., 2016	?	+	-	+	-	-	?	+	+	+	5
Wu et al., 2010	+	+	-	+	-	-	?	+	+	+	6
Eshaghian et al., 2018	+	+	-	+	-	-	?	+	+	+	6
Erika et al., 2020	?	+	-	+	-	-	?	+	+	+	5
Khodarahmi et al.,2020	+	+	-	+	-	-	?	+	+	+	6
Zhang et al., 2013	?	+	-	+	-	-	?	+	+	+	5
Wu et al., 2008	+	+	-	+	-	-	?	+	+	+	6
Karina et al., 2008	+	+	-	?	-	-	?	+	+	+	5
Zhao et al., 2014	+	+	-	+	-	-	?	+	+	+	6
Qin et al.,2018	+	+	-	+	-	-	?	+	+	+	6
Lu et al., 2017	+	+	-	+	-	-	?	+	+	+	6
Tu et al., 2012	+	+	-	+	-	-	?	+	+	+	6
Abd-Allah et al., 2016	+	+	-	+	-	-	?	+	+	+	6
Morsy et al.,2012	+	+	-	+	-	-	?	+	+	+	6
Lee et al., 2016	?	+	-	?	-	-	?	+	+	+	4
Fu et al., 2008	+	+	-	?	-	-	?	+	+	+	5
Zhao et al., 2018	+	+	-	+	-	-	?	+	+	+	6
Kabirifar et al., 2018	+	+	-	+	-	-	?	+	+	+	6
Barta et al., 2015	+	+	-	+	-	-	?	+	+	+	6
George et al., 2006	+	+	-	?	-	-	?	+	+	+	5
Kyung et al., 2018	+	+	-	+	-	-	?	+	+	+	6
Abo-Zaid et al., 2020	+	+	-	?	-	-	?	+	+	+	5

A. sequence generation; B. baseline characteristics; C. allocation concealment D. random housing; E. blinding (caregivers/investigators) F. random for outcome assessment; G. blinding (outcome assessor) H. incomplete outcome data; I. selective outcome reporting; J. other biases

Supplementary 3

Subgroup analysis

1. subgroup analysis based on species

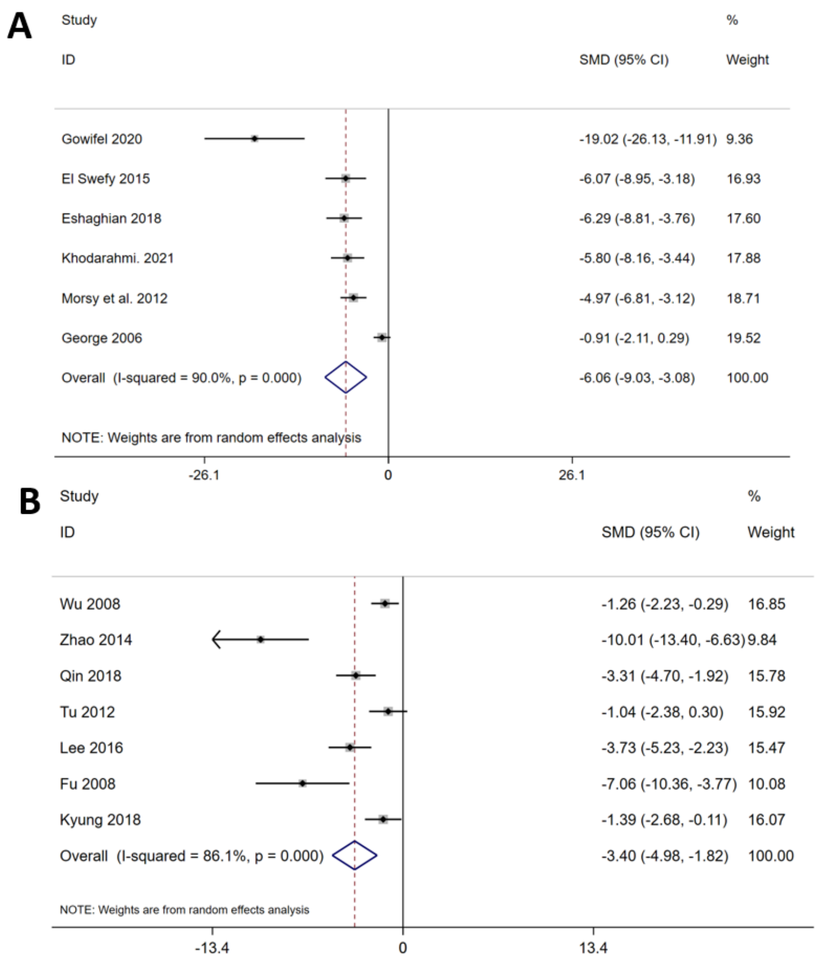

Figure 1. Forest plot: Subgroup analysis of pooled estimates of AST based on species.

(A) Wistar rats; (B) Sprague-Dawley rats.

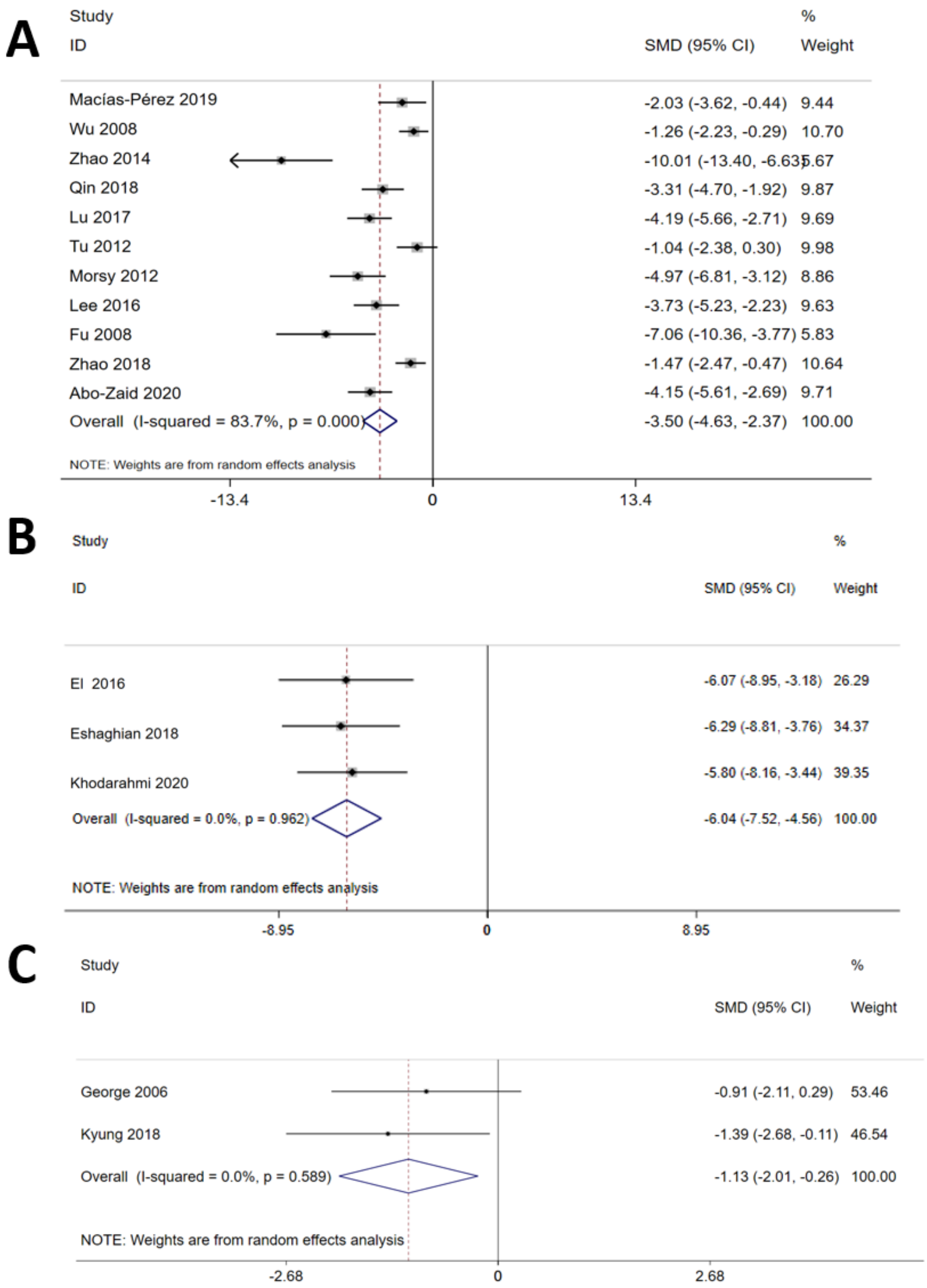

Figure 2. Forest plot: Subgroup analysis of pooled estimates of AST based on animal models.

(A) CCl4; (B) BDL; (C) NDMA.

2. Subgroup analysis of Subgroup analysis of ALT

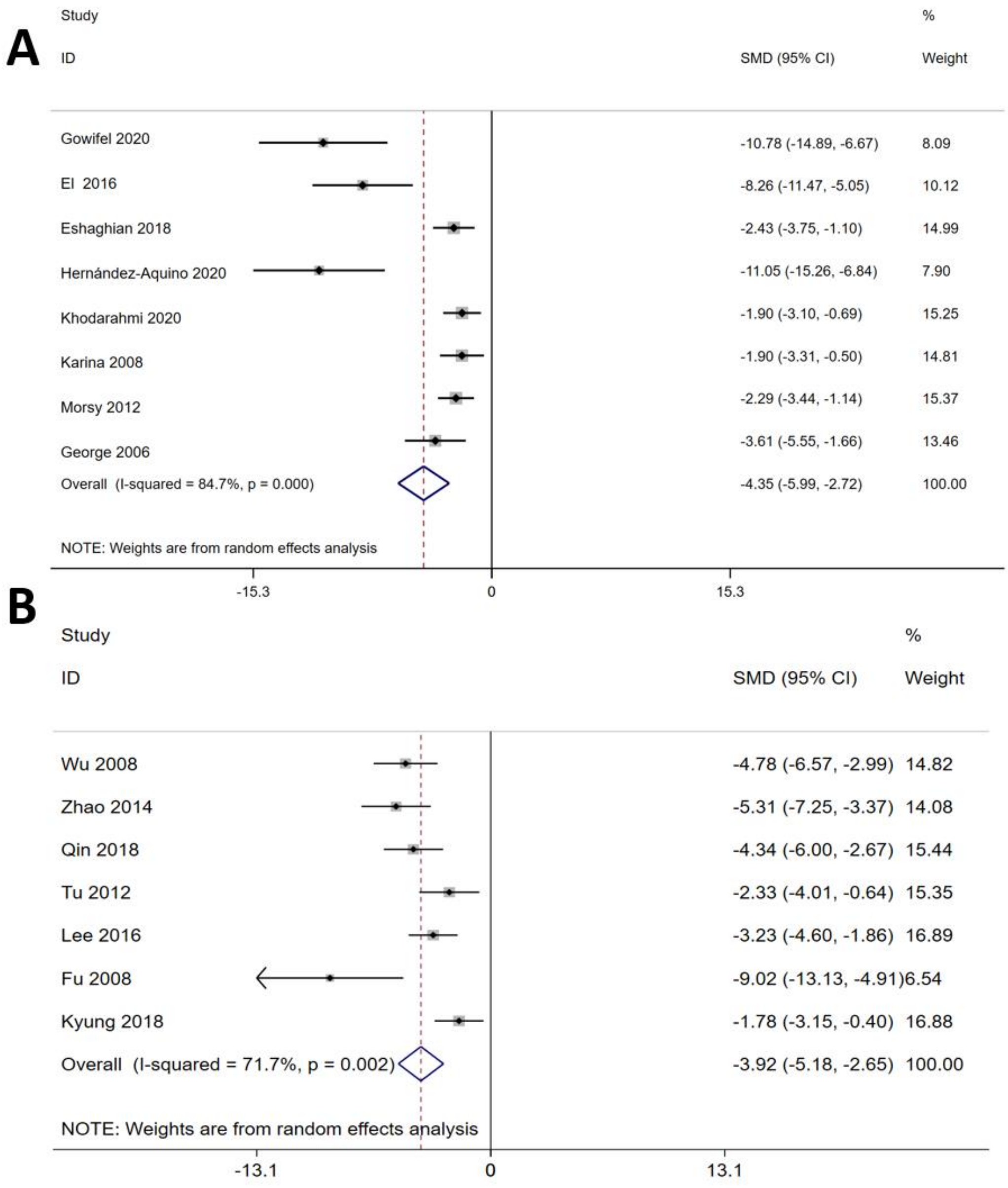

Figure 3. Forest plot: subgroup analysis of pooled estimates of ALT based on species.

(A)Wistar rats; (B) Sprague-Dawley rats.

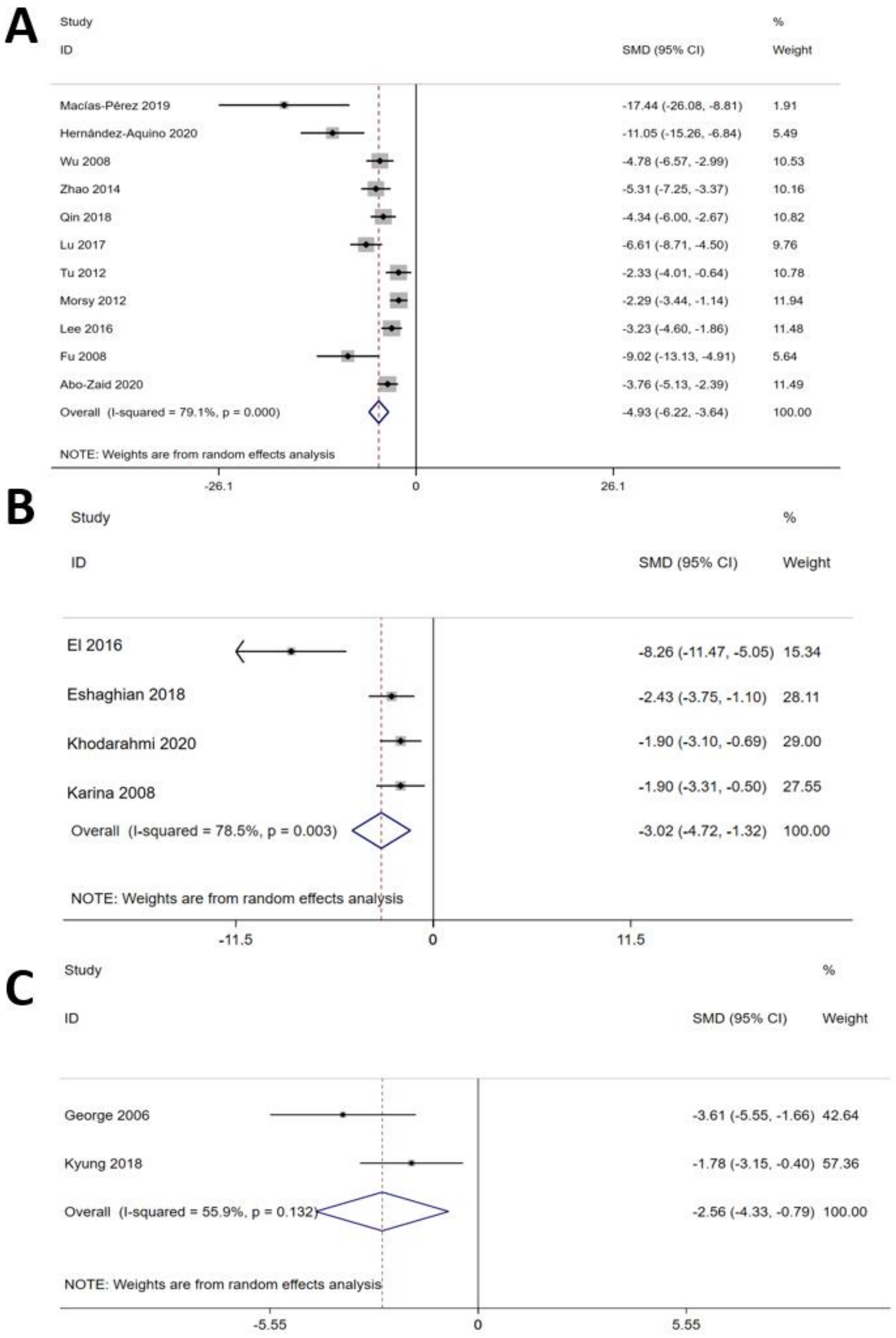

Figure 4. Forest plot: subgroup analysis of pooled estimates of AST based on animal models.

(A)CCl4; (B) BDL; (C)NDMA.
